# 3D Traction Forces in Cancer Cell Invasion

**DOI:** 10.1371/journal.pone.0033476

**Published:** 2012-03-30

**Authors:** Thorsten M. Koch, Stefan Münster, Navid Bonakdar, James P. Butler, Ben Fabry

**Affiliations:** 1 Department of Physics, University of Erlangen-Nuremberg, Erlangen, Germany; 2 Molecular Integrative Physiological Sciences Program, Harvard School of Public Health, Boston, Massachusetts, United States of America; 3 Division of Sleep Medicine, Department of Medicine, Harvard Medical School and Brigham and Women's Hospital, Boston, Massachusetts, United States of America; University of Bristol, United Kingdom

## Abstract

Cell invasion through a dense three-dimensional (3D) matrix is believed to depend on the ability of cells to generate traction forces. To quantify the role of cell tractions during invasion in 3D, we present a technique to measure the elastic strain energy stored in the matrix due to traction-induced deformations. The matrix deformations around a cell were measured by tracking the 3D positions of fluorescent beads tightly embedded in the matrix. The bead positions served as nodes for a finite element tessellation. From the strain in each element and the known matrix elasticity, we computed the local strain energy in the matrix surrounding the cell. We applied the technique to measure the strain energy of highly invasive MDA-MB-231 breast carcinoma and A-125 lung carcinoma cells in collagen gels. The results were compared to the strain energy generated by non-invasive MCF-7 breast and A-549 lung carcinoma cells. In all cases, cells locally contracted the matrix. Invasive breast and lung carcinoma cells showed a significantly higher contractility compared to non-invasive cells. Higher contractility, however, was not universally associated with higher invasiveness. For instance, non-invasive A-431 vulva carcinoma cells were the most contractile cells among all cell lines tested. As a universal feature, however, we found that invasive cells assumed an elongated spindle-like morphology as opposed to a more spherical shape of non-invasive cells. Accordingly, the distribution of strain energy density around invasive cells followed patterns of increased complexity and anisotropy. These results suggest that not so much the magnitude of traction generation but their directionality is important for cancer cell invasion.

## Introduction

Cell migration through a connective tissue matrix is an important part of normal physiological function, for example during wound healing, but is also a hallmark of aberrant behavior seen in cancer cell invasion through connective tissue. Cell migration on planar 2D matrices, such as on a common plastic tissue culture dish, has been described as a cyclic process involving polarization, protrusion formation at the leading edge, traction generation by the cells' acto-myosin machinery, and retraction at the rear end of the cell [1]. Inertial and viscous drag forces are negligible, and cell tractions are needed only for cell spreading and for overcoming integrin-mediated adhesive forces.

Cell migration through a dense 3D network of extracellular matrix proteins, in contrast to 2D migration, is possible only when the cell generates sufficient tractions to overcome the steric hindrance of the surroundings [2]. The migration speed of cells in a 3D matrix correlates with the maximum matrix displacements, which are indicative of the traction forces that these cells exert [3]. Cells in which acto-myosin contraction is inhibited are unable to migrate through dense 3D matrices [4]. These findings lead to the hypothesis that cancer cells that generate high tractions are more invasive than cells with lower tractions. In this study we present a method by which these tractions can be quantified, and we test this hypothesis in differently invasive carcinoma cell lines.

2D cell tractions can be measured by observing the displacements of beads embedded in a planar flexible gel substrate on which the cells are cultured. Mathematically, this is an ill-posed problem to which several approaches have been successfully developed [5]. Early approaches inverted the relationship between displacements and tractions of a cell on a semi-infinite elastic halfspace using regularization [6] or Fourier [7] methods. Those approaches were recently refined to account for a finite thickness of the elastic substrate [8], [9], [10], to improve the spatial resolution [11], and to include traction components normal to the substrate [12].

Beads can also be dispersed in 3D cell culture systems to estimate cellular contractility during migration [13], [14]. Using this approach, 3D cell tractions and their spatial distribution were recently measured for the first time by extending the ideas of 2D traction microscopy to the third dimension [15]. This novel method is technically and computationally involved, however, and requires a synthetic polymer gel with linear elastic properties as a 3D matrix. Here we present a method to quantify 3D contractility of cells in virtually any biopolymer network using a standard fluorescence microscope. The method is computationally efficient and robust against measurement noise. Instead of computing the full 3D traction map of the cell, we measure the strain energy and its density distribution in the 3D extracellular matrix around isolated cells. This method gives a scalar measure for the total cellular contractility. The source code of all necessary programs to carry out the measurements is provided in Supporting Information File S1.

We have used this method to compare the contractility of several tumor cell lines with different abilities to invade a dense collagen network. We show that high contractility is required but is not sufficient for invasion through a dense 3D network; some non-invasive tumor cells can also generate high contractile forces but lack the ability to direct those forces to drive their locomotion.

## Results

### Measurement of strain energy

To measure the strain energy that cells expend to deform their three-dimensional surroundings, cells are either embedded within monomeric collagen prior to polymerization, or are cultured on a collagen gel surface and then allowed to spontaneously invade into the collagen bulk. Under both conditions, the cells spread and extend into the porous collagen structure and exert traction forces that deform the gel. The energy thus expended is stored as elastic strain energy in the gels. By measuring the deformation of the collagen gels, it is possible to compute the strain energy if the elastic mechanical properties of the collagen matrix are known.

Gel deformations are determined from measuring the positions of fluorescent marker beads that are embedded throughout the gel. We record optical sections every 2 µm through the entire thickness (∼500 µm) of the gels (Movie S1). Using a difference-with-interpolation algorithm, we can resolve bead displacements with sub-pixel resolution (Supporting Information Text S1). The accuracy of the displacement measurement for Ø 1 µm beads at 10× magnification is 22 nm in the x-y-plane and 130 nm along the optical axis.

In this study we use reconstituted collagen gels that form spontaneously during self-assembly of collagen fiber networks (Fig. S1a). At a collagen concentration of 2.4 mg/ml, these networks form a porous structure with an average pore size of 1.3 µm [16]. On a length scale larger than 10 µm, the network morphology can be regarded as homogeneous and isotropic [16]. The average distance between the embedded fluorescent beads is ∼24 µm (Fig. S1b), which is much larger than the pore size so that we can use a continuum approximation to describe the mechanical properties of the collagen network. For a validation of this approximation see Supporting Information Text S1 (Fig. S8, S9). Measurements with a cone-plate rheometer revealed a predominantly elastic response of the collagen network (Fig. S1d). Collagen gels also exhibit non-linear behavior with prominent strain stiffening (Fig. S1c), but for strains of up to 5%, the material properties can be approximated by a linear elastic behavior with a shear modulus *G* of 118 Pa. We remark on this approximation in Discussion.

The strain energy of cells in collagen gels is calculated from bead displacements between the deformed and undeformed state of the gels. The undeformed, force-free state of the collagen is obtained by treatment of the cells with 4 µM cytochalasin D which disrupts the actin cytoskeleton and suppresses force generation (Movie S2). The bead positions of the undeformed gel are used for tessellating the extracellular microenvironment and serve as the corner nodes of tetrahedral finite elements (Fig. S3). The bead displacements then give rise to a strain energy in each finite element. The strain energy of an element, normalized by the element volume, gives the strain energy density (Supporting Information Text S1).

This method of deriving the material strain from a tessellation of measured positions is sensitive to measurement noise, and care must be taken to suppress their contributions to the strain energy results. This effect is due to the fact that strain energy is a quadratic function of the displacement field. Therefore, the expectation value of noise contributions is nonzero even if the noise fluctuates around zero. Erroneous strain energies from noise can be largely avoided by identifying and eliminating tetrahedral elements that are flat in the sense of planar near-degeneracy (Fig. S4, S5), and by subtracting an experimentally determined strain energy baseline caused by bead displacement noise (Fig. S6 and Supporting Information Text S1).

**Figure 1:**
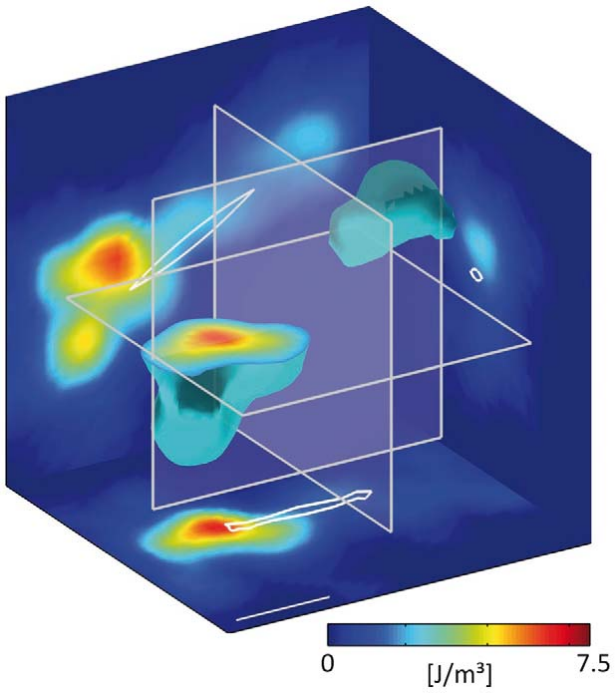
Strain energy density around a breast carcinoma cell. Strain energy density around an elongated MDA-MB-231 breast carcinoma cell embedded ∼400 µm deep in a collagen gel. An isosurface of strain energy is shown with cuts projected to the coordinate planes as indicated by the transparent planes. The strain energy density is highest in close proximity of the cell poles and decays rapidly in all directions away from the cell. Integration of strain energy density gives the total strain energy and was 3.7 pJ in this example. The scale bar is 50 µm.

The strain energy density is distributed in complex patterns in the volume surrounding the cell (Fig. 1). In general, the strain energy density is highest in close proximity of the cell, in particular near the cell poles, and falls quickly to negligible levels in all directions away from the cell. The total strain energy is obtained by integrating the strain energy density over a large volume around the cell. Our method includes the cell volume itself, assuming it to have an elastic modulus similar to that of the extracellular matrix – we remark on this assumption in Discussion.

### Strain energy of invasive and non-invasive carcinoma cells

**Figure 2:**
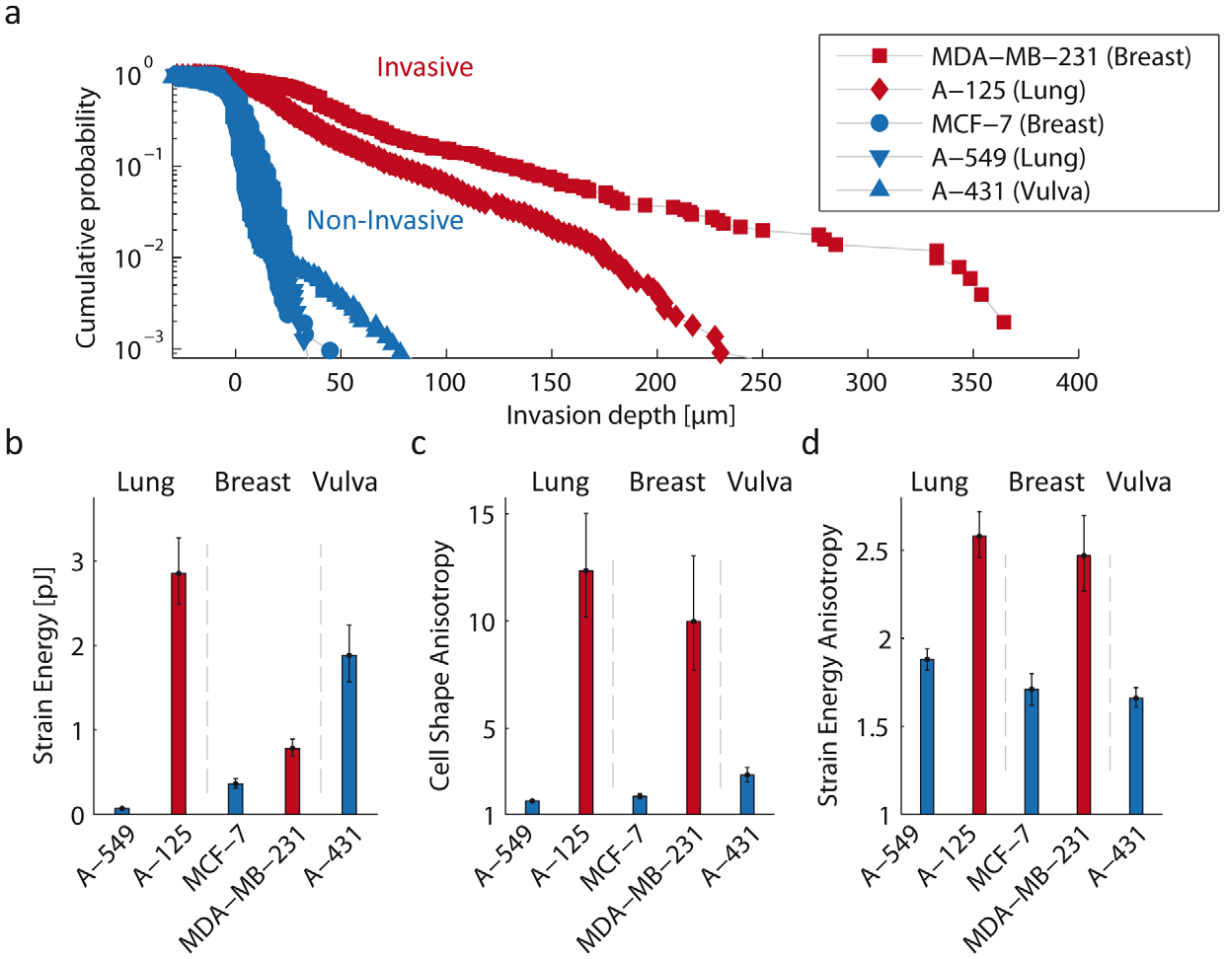
Strain energy and anisotropy of non-invasive and invasive cells. a: Invasion profiles of A-125 and A-549 lung, MDA-MB-231 and MCF-7 breast and A-431 vulva carcinoma cells. Cells were plated on the surface of collagen gels and allowed to spread and invade for 3 days. The invasion profile vs. gel depths describes the cumulative probability of finding a cell below a given depth. Invasive cells were characterized by their ability to invaded deep into the gels. b: Strain energy of non-invasive and invasive carcinoma cells. Invasive lung (n = 36) and breast (n = 33) carcinoma cells generate significantly higher strain energies compared to non-invasive lung (n = 49) and breast (n = 31) carcinoma cells. Non-invasive vulva carcinoma cells (n = 35), however, generate the largest strain energy of all cell lines tested (Fig. S10). c: Anisotropy of cell shape. Non-invasive cells are significantly rounder compared to invasive cells. d: Anisotropy of the strain energy density is significantly higher in invasive compared to non-invasive cells. Because the strain energy and anisotropy values from different cells follow a log-normal distribution (Supporting Information Text S1, Fig. S10, S11, S12), the geometric mean ± geometric standard error are shown in Fig. b–d.

To test the hypothesis that invasive carcinoma cells generate higher tractions compared to non-invasive cells, we measured the 3D strain energy of invasive cells (A-125 lung and MDA-MB-231 breast carcinoma cells), and of non-invasive cells (A-549 lung, MCF-7 breast and A-431 vulva carcinoma cells (ATCC-LGC-Promochem, Wesel, Germany)). The invasiveness of these cell lines was determined by seeding the cells on a thick collagen gel and allowing them to invade into the gels. After three days, we measured the invasion profiles as the spatial distribution of cell density at various gel depths [17]. Most cells from the non-invasive cell lines were unable to invade into the bulk of the collagen gel, and the few cells that did invade remained close to the surface (Fig. 2a). Invasive cells, in contrast, were able to invade deep into the gels (Fig. 2a).

For strain energy measurements, the cells were embedded in the gels prior to polymerization, and isolated cells in the central region of the gel were selected for measurements. Both invasive lung and breast carcinoma cells generated considerably higher 3D tractions than non-invasive lung and breast carcinoma cells, as reflected in the significantly higher strain energy of those cells (Fig. 2b). High contractility, however, did not always correlate with cell invasiveness. We found that non-invasive A-431 vulva carcinoma cells were surprisingly contractile (Fig. 2b). Furthermore, invasive lung carcinoma cells were 4-fold more contractile than invasive breast carcinoma cells, yet they were less invasive (Fig. 2a).

**Figure 3:**
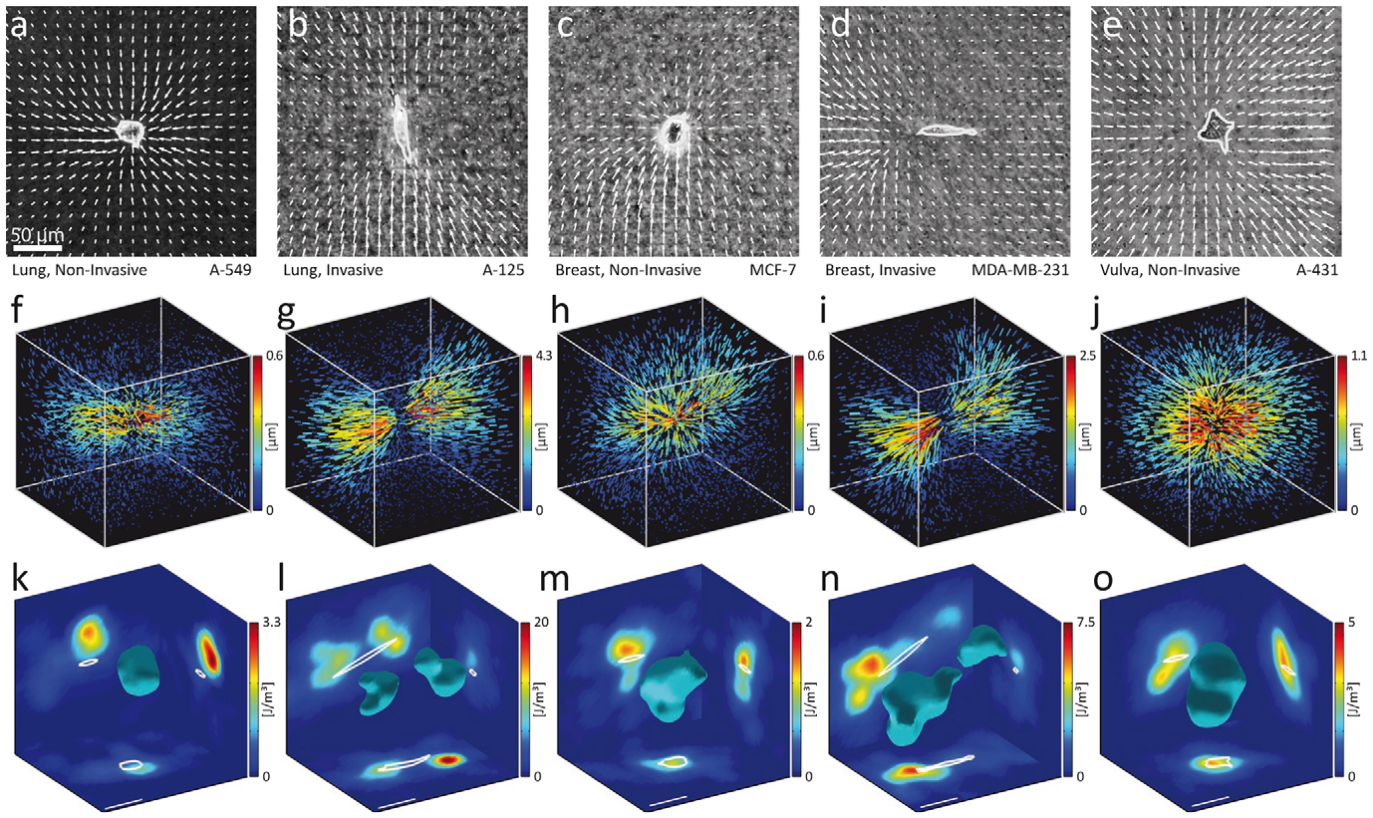
Displacements and strain energy density around non-invasive and invasive cells. a–e: Displacement fields (projected to the x-y plane, normalized to the largest displacements) around invasive and non-invasive carcinoma cells. Non-invasive cells contract the gels more isotropically. Invasive cells generate highly anisotropic displacement fields with large displacements at the cell poles and a region of comparatively small displacements near the cell center. f–j: 3D displacement fields of the same cells as shown in a–e. k–o: Strain energy density around the same cells as shown in a–e. A closed isosurface of strain energy density (30% of maximum value) is shown with cuts projected to the coordinate planes. The strain energy density is distributed more isotropically around non-invasive cells with almost spherical isosurfaces, and is distributed more anisotropically around invasive cells with complex isosurface shapes.

We noted that invasive carcinoma cells from both the lung and the breast had an elongated spindle-like morphology, whereas non-invasive cells had a rounder shape (Fig. 3a–e). We quantified cell shape anisotropy by the ratio of maximal to minimal eigenvalues of the second moments of the cell contour. A value of 1 corresponds to a circular shape, and increasing values to more elongated shapes. Invasive cell lines showed a significantly higher cell shape anisotropy compared to non-invasive cells (Fig. 2b).

Cell shape anisotropy is accompanied by an anisotropic distribution of cellular tractions and strain energy around the cell (Fig. 2c). We quantified the strain energy anisotropy again by the ratio of maximal to minimal eigenvalues of the second moments of the strain energy density around a cell. The strain energy was distributed more anisotropically around invasive cells whereas non-invasive cells contracted the gel more isotropically in all directions.

**Figure 4:**
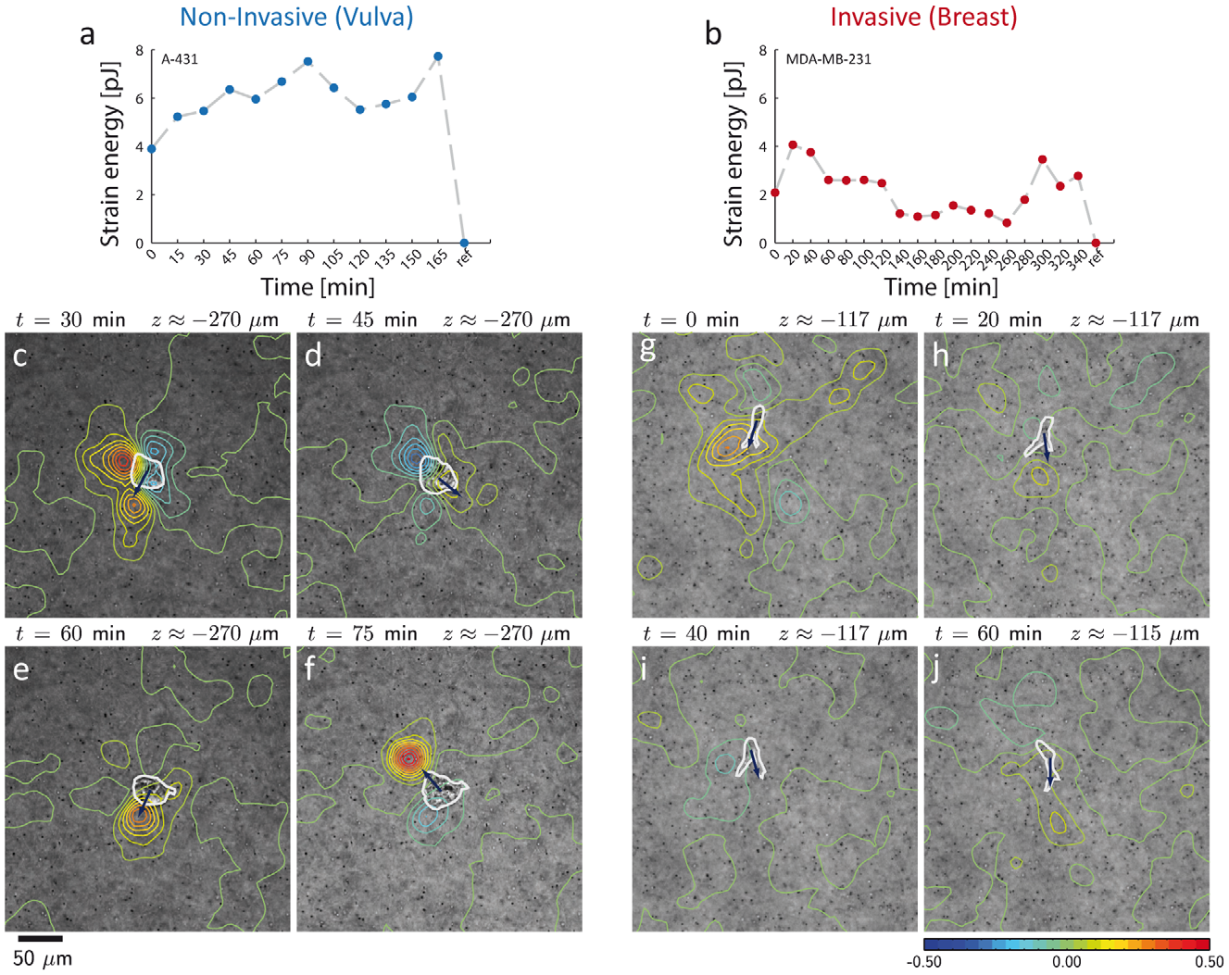
Time-lapse measurements of non-invasive and invasive cells. a: Time course of the total strain energy around a non-invasive vulva carcinoma cell (A-431) in a 3D collagen gel. The zero energy reference was measured after cytochalasin D-induced tension release. The total strain energy fluctuates by about ±25% around a high mean value. b: Time course of the total strain energy around an invasive breast carcinoma cell (MDA-MD-231) in a 3D collagen gel. The strain energy shows large fluctuations of about ±50% around the mean. c–j: Time series of brightfield images of a non-invasive vulva carcinoma cell (A-431) (c–f) and an invasive MDA-MB-231 breast carcinoma cell (g–j) in a 3D collagen gel. Superimposed are contour lines showing relative changes in strain energy density between two subsequent time steps. Blue colors indicate regions where tension is relaxed between successive time steps, and red colors indicate regions where tension is increased. The cell shape is outlined in white for clarity. Dark blue arrows indicate the direction of cell movement between the two time steps (see Fig. S13, S14, S15, S16, and S17 and Movies S3 and S4 for more details).

Further insight into the role of anisotropic force distribution during carcinoma cell invasion can be gained by combining strain energy density measurements with time-lapse recording. Invasive MDA-MB-231 breast carcinoma cells adapt their contractile states (Fig. 4b) in concert with their migration through the gel. Time-lapse images of a representative cell show that directional changes in the local strain energy density precede the change in migration direction (Fig. 4g–j; see Fig. S15, S16 and S17 and Movie S4 for complete data set). A contrasting data set shows a time-lapse measurement of a highly contractile but non-invasive vulva carcinoma cell. The cell's contractile behavior (Fig. 4a) is also not static, although the fluctuations are smaller compared to invasive cells (Fig. 4b). Importantly, however, the cell is not able to generate an anisotropic distribution of tractions and strain energy over prolonged periods of time (Fig. 4c–f; see Fig. S13, S14 and Movie S3 for complete data set), and thus the net movement of the cell is negligible (Supporting Information Text S1).

## Discussion

The strain energy that cells exert on their surroundings is a robust scalar measure of cell tractions in a 3D biopolymer matrix such as a collagen gel. The method is computationally efficient; the analysis of approximately 10,000 bead displacements between two consecutive image stacks together with the evaluation of the resulting strain energy density map takes only a few minutes on a standard desktop computer. Moreover, the method can be readily implemented on any fluorescence microscope with a motorized z-focus.

The method is based on several simplifying assumptions. First, we neglect the fibrous network structure of the collagen gel and describe it as a continuum. Gel deformations are sampled at the bead locations, and the strain field between the beads is computed by a tri-linear interpolation. At the length scale of an average bead separation of 24 µm (Fig. S1b), this approach is a good approximation but underestimates the true strain energy (Fig. S7).

Second, we assume linear elastic material properties and ignore the strain stiffening response of the matrix. This leads to an underestimation of the absolute strain energy especially for highly contractile cells in highly non-linear matrices. This is not an intrinsic shortcoming of our method, however. The linear stiffness matrix of the collagen can be easily replaced by a non-linear constitutive model for the collagen gels (Supporting Information Text S1). Alternatively, the linear regime of fibrous biopolymer networks can be extended by the addition of a hydrogel [18].

Third, the cell's mechanical properties are assumed to be the same as the surrounding collagen gel, and the strain energy is computed over the whole volume, i.e. the collagen surrounding the cell and the volume occupied by the cell itself. However, as the cell volume is small – typically less than 3 tetrahedral elements – the resulting error is negligible.

An inherent limitation of 3D traction assays is that cells can locally degrade, synthesize, and crosslink the extracellular matrix and thereby alter the matrix mechanical properties. To minimize global matrix remodeling, we mixed only a small number of cells with the gel before polymerization. Any local changes in matrix mechanical properties can then be reasonably assumed to be limited to a volume around the cell that is small compared to the typical gel volume with high strain energy densities.

While our technique cannot provide a detailed traction map over the cell surface, it offers several advantages in comparison with recently developed approaches. The distribution of strain energy density is resolved with sufficient spatial resolution to distinguish areas of high and low contractility along the cell shape. Together with the displacement field, this is adequate information about 3D cellular contractility for many biological questions. Moreover, the strain energy distribution can be integrated over the matrix volume surrounding the cell to obtain a single, scalar and biologically relevant measure of cell contractility – the total strain energy. Most importantly, our technique can be used to measure 3D cellular contractility in any biopolymer network. If the biopolymer network is non-linear but a linear approximation is used for the computation of the strain energy, there will be inevitably some error in the strain energy densities in those areas where the cell-induced strain reach the non-linear regime. However, this error is not degenerative in the sense that it occurs only once at each spatial position of an element and does not propagate to neighboring elements (as is the case with tractions that are highly sensitive to distant displacements and matrix non-linearities).

Our data demonstrate that invasive cells exert considerable forces on their environment. The strain energy exerted by MDA-MB-231 breast carcinoma cells in a soft (118 Pa) 3D gel was approximately 10 fold higher than the values that have been measured for cells cultured on stiff (4.8 kPa) polyacrylamide gels [17]. This is surprising, as many cell types become more contractile on stiffer matrices [19]. We speculate that the dimensionality of the surrounding matrix may play an important role for the ability of cells to generate tractions.

Large contractile forces, however, are not sufficient for cell invasion. Our data show that non-invasive carcinoma cells are able to generate very high strain energies. For efficient cell invasion, it is important that the cell tractions are used efficiently by directing them along a particular migration path. Consistent with this view, we found that the cell shape and the distribution of strain energy density are highly anisotropic in invasive cells, but nearly isotropic in non-invasive cells. A connection between anisotropic cell shape and invasive behavior has been previously described in cells after epithelial-to-mesenchymal transition [20], and it has been speculated that the mechanism for increased invasiveness in these cells is an anisotropic force distribution [21] and the result of spatio-temporal coordination of protrusion, attachment, traction generation, and release [22], [23]. The findings presented here support this view and point to the importance of shape anisotropy and polarity in migrating cells. Our technique offers a tool to study these processes in a 3D environment.

## Methods

### Three-dimensional collagen assay

Rat tail collagen (Collagen R, Serva, Heidelberg, Germany) and bovine skin collagen (Collagen G, Biochrom, Berlin, Germany) were mixed at a ratio of 1∶1. We then added 10 vol% of sodium bicarbonate (23 mg/ml), orange fluorescent latex beads with 1 µm diameter (FluoSpheres®, Invitrogen, F8820) and 10 vol% of 10× DMEM (Biochrom). The solution was neutralized with 1N sodium hydroxide. Approximately 2000 cells were mixed in 1.2 ml collagen solution and added to a 35 mm culture dish. The solution was polymerized at 37°C, 95% humidity and 5% CO_2_ for 1 h. Polymerized collagen gels had a thickness of approximately 500 µm.

### Optical sections

To quantify 3D gel deformation, we repeatedly recorded equidistant optical z sections (2 µm apart) through the entire gel thickness using a motorized inverted fluorescence microscope (DMI6000B, 20× objective, NA = 0.4, 0.5× video coupler, Leica Microsystems, Germany) equipped with a CCD camera (ORCA ER, Hamamatsu Photonics, Germany). The microscope was additionally fitted with a custom-made stage incubator for keeping the cells at 37°C, high humidity and 5% CO_2_. The measurement process was automated with custom software. To determine the deformation of the collagen gels, at least two image stacks were recorded. The first image stack corresponded to the deformed state of the gel. The second image stack was recorded after the cells were treated for at least 10 min with cytochalasin D (4 µM) to disrupt the actin cytoskeleton and to obtain the force-free, undeformed state of the gel.

### 3D positions of beads

The 3D displacement of each bead was determined using a 3D difference-with-interpolation algorithm applied to the two image stacks. The bead positions in the first image stack are identified with a simple intensity threshold. A subvolume of 4.5 µm×4.5 µm×14.0 µm in the x-, y and z-direction was then cut out around each bead. The position of the same bead in the second image stack was then determined by shifting the subvolume from the first stack relative to the second stack until the squared differences of the pixel intensities were minimized (Fig. S2). Sub-pixel accuracy was achieved by tri-linear interpolation of pixel intensities. With this method, the 3D displacements of beads can be measured with an accuracy of 22 nm in the x- and y- direction, and 130 nm in the z-direction.

### Measurement of strain energy

To compute a continuous strain field from discrete bead positions and their displacements, we use a finite element approximation of the gel where the bead positions serve as the nodes of linear tetrahedral elements. The finite element mesh was obtained by Delaunay tessellation. The node displacements are then given by the bead displacements between two image stacks. Each element was assigned with isotropic linear elastic material properties (shear modulus 118 Pa, Poisson's ratio 0.35). The strain energy of each element was then calculated as a product of the stiffness matrix with the displacement components (Supporting Information Text S1).

### Noise elimination

The strain energy computation from displacement data is sensitive to measurement noise, but it has only an additive effect that can be largely corrected for by simple subtraction. The degree to which an individual tetrahedral element is affected depends on its volume and its shape. The smaller the element and the flatter its shape, the more prone it is to spurious strain energy results. The shape of an element can be quantified with an appropriate shape measure. Here we use a ratio of element volume to the sum of its cubed edge lengths. Normalized by 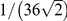, this value ranges from 0 (corresponding to maximally degenerate elements) to 1 (the value of a regular tetrahedron with all 6 edges of equal length). Elements with a shape factor smaller than 0.5 are disproportionately sensitive to displacement noise and are therefore excluded from the computation. The gaps in the strain energy density field by those missing elements are filled by nearest neighbor interpolation. The strain energy error of the remaining elements can be calculated from knowledge of the displacement errors and is subtracted from the results.

### Tumor cell invasion assay

Collagen gels were prepared as described above, but without the incorporation of fluorescent beads. 25,000 cells were seeded on top of the collagen matrix and cultured for 72 h. At this time period, differences in the invasiveness of cells were clearly visible. After fixation with 2.5% glutaraldehyde solution in PBS and Hoechst 33342 nuclei staining, the number of invaded cells and their invasion depth were determined in 50 non-overlapping fields of view around the center of the gels. This measurement was automated by custom-made software that locates the nuclei of cells in stacks of images recorded at 2 µm interval through the entire thickness of the gels.

### Cell shape anisotropy

To quantify cell shape we employed an analysis of second moments on 2D projections of the cell. Each cell was outlined manually with a closed polygon in the phase contrast image at the z-position of greatest sharpness as defined by a Tenenbaum gradient measure [24]. The closed polygon was then used to create a binary image of the cell, 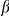. A matrix of second moments can then be calculated as 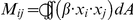, where the x- and y- pixel coordinates 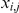 are centered at the center of mass of 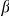. Cellular shape anisotropy is defined by the ratio of the maximum to minimum eigenvalue of 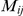. A perfectly round cell would thus have an anisotropy value of 1, and increasing values denote increasingly anisotropic shapes such as elongated spindle-like forms.

### Anisotropy of strain energy density distribution

To characterize the anisotropy of the strain energy density distribution around a cell, we calculate the second moments of the strain energy density 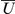
as 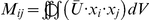, where the x-, y- and z-coordinates 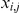 are centered at the center of mass of the strain energy density. The index of anisotropy of the strain energy density distribution is then defined as the ratio of maximum to minimum eigenvalue of the second moments. Thus, a perfectly isotropic distribution of strain energy density would result in a value of 1, and increasing values in this index are indicative of distributions with increasing anisotropy.

## Supporting Information

Figure S1 Properties of collagen gels. a) Confocal section through a collagen gel. b) Distribution of bead-bead distances. c) Stress-strain relationship of the collagen network measured in a cone-plate rheometer during a strain ramp (speed: 1%/s). The behavior is approximately linear for strains below 5%; d) Frequency response measured in a cone-plate rheometer. The amplitude of sinusoidal oscillations was 5%. The storage modulus G′ is 118 Pa at a frequency f of 1 Hz. Storage modulus G′ and loss modulus G″ increased weakly with frequency according to a power-law with exponent 0.08, indicative of predominantly elastic behavior.
(TIF)

Figure S2 Intensity distribution in a subvolume around a bead at two distinct time points. To track the bead position, the subvolume at t = 2 is shifted until a best match with the subvolume at t = 1 is achieved.
(TIF)

Figure S3Linear interpolation of displacements. The displacements at any location within a tetrahedral element are linearly interpolated with the use of shape functions according to Eq. (5). These shape functions are defined in a fixed 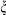, 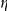, 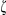 coordinate system of a parent tetrahedral element shown on the left. The map in Eq. (5) is then fully defined by the 4 corner nodes (i.e. the bead positions) of a tetrahedron in the global 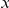, 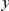, 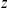 coordinate system.(TIF)

Figure S4 Shapes of tetrahedral elements. a–c: Tetrahedral elements of various shapes with different shape quality factors q. Elements with small shape factors have a highly degenerate geometry, e.g. they are flat (a). As the shape factors increase, the elements look increasingly like a regular tetrahedron (b and c) d: Distribution of shape quality factors in a typical collagen gel.
(TIF)

Figure S5Tetrahedral shape influences erroneous strain energy density. Bead position noise causes erroneous strain energy densities (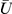 normalized to the expected value 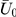 of a tetrahedron with a shape factor of 0.1) with a log-normal probability density distribution. The expected value and the width of the distribution depend on the shape of the tetrahedra. As the shape quality factor gradually decreases, the distribution widens and the expected value increases. The inset shows the geometric mean of the erroneous strain energy as a function of the shape quality factor q. For values of q<0.5, the tetrahedral elements become excessively sensitive to noisy displacements.(TIF)

Figure S6 Noise correction of strain energy density. a: strain energy density distribution around an A-125 lung carcinoma cell without any correction, b: after eliminating tetrahedral elements with shape quality factor q<0.5, c: after additional noise subtraction according to Eq. (15). d: Integration of strain energy density over increasingly larger volumes shows the importance of shape and noise correction for recovering the strain energy. Here, the solid lines correspond to strain energy measurements of the cell shown in a–c; the dashed lines to the strain energy measured in a gel without cells.
(TIF)

Figure S7 Strain energy convergence. Strain energy in a fixed volume around two representative cells evaluated on a subset of beads, i.e. the number of beads in an experimental data set was systematically reduced (a, b, and c show resulting finite element meshes) to obtain the curves in d.
(TIF)

Figure S8 Displacements and strain energy density around a deflected superparamagnetic bead. a: Measured (blue) and fitted (red) projected displacements of fluorescent beads embedded in a collagen gel in the vicinity of a superparamagnetic bead. The gel was deformed with a force of 58 nN acting on the magnetic bead. b: Measured displacements of the fluorescent beads in the collagen drop between the force-on and force-off states. c: Corresponding strain energy density distribution around the magnetic bead indicated by the white star. The direction of the applied force is shown by the white line.
(TIF)

Figure S9 Experimental verification of the strain energy measurement by an indentation experiment with a steel ball. a: Finite Element simulation of the experiment. b: Measured displacements projected on a cylindrical coordinate system with the z axis going through the center of the steel ball. c: The strain energy was evaluated in a donut shaped subvolume as indicated by the red lines in b).
(TIF)

Figure S10 Cumulative probabilities of total strain energy for various carcinoma cell lines. Each data point corresponds to a measurement from a different cell. The lines are cumulative probabilities from a best-fit log-normal distribution. With the exception of A-431 cells, cellular strain energy data follow a log-normal distribution.
(TIF)

Figure S11 Cumulative probabilities of cell shape anisotropy for various carcinoma cell lines. Each data point corresponds to a measurement from a different cell. The lines are cumulative probabilities from a best-fit log-normal distribution.
(TIF)

Figure S12 Cumulative probabilities of strain energy density anisotropy for various carcinoma cell lines. Each data point corresponds to a measurement from a different cell. The lines are cumulative probabilities from a best-fit log-normal distribution.
(TIF)

Figure S13 Time-lapse measurement of a non-invasive vulva carcinoma cell. Time series of brightfield images at the location of a non-invasive vulva carcinoma cell (A-431) embedded in a 3D collagen gel. Superimposed are contour lines encoding relative changes in strain energy density between two subsequent time steps, indicating how the distribution of strain energy density shifts around the proximity of the cell during the measurement. The cell shapes at the first and second time step are outlined in white and dark blue, respectively, to indicate the changes in cell position.
(TIF)

Figure S14 Continuation of Fig. S13.
(TIF)

Figure S15 Time-lapse measurement of an invasive breast carcinoma cell. Time series of brightfield images at the location of an invasive breast carcinoma cell (MDA-MD-231) embedded in a 3D collagen gel. Superimposed are contour lines encoding relative changes in strain energy density between two subsequent time steps, indicating how the distribution of strain energy density shifts around the proximity of the cell during invasion. The cell shapes at the first and second time step are outlined in white and dark blue, respectively, to indicate the changes in cell position.
(TIF)

Figure S16 Continuation of Fig. S15.
(TIF)

Figure S17 Continuation of Fig. S16.
(TIF)

Movie S1 Sequence of optical sections through the entire thickness of a collagen gel. Phase contrast images are shown with the fluorescence signal superimposed in green. An isolated A-125 lung carcinoma cell was embedded in its center. Fluorescent marker beads were embedded throughout the gel and were used to measure gel deformations.
(MOV)

Movie S2 Relaxing the cells. An isolated A-125 lung carcinoma cell embedded in a collagen gel (shown at constant depth) before and after treatment with cytochalasin D (4 µM). The pharmacological treatment relaxed the cell and recovered the undeformed, force-free state of the gel, which can be seen in the displacements of the marker beads around the cell.
(MOV)

Movie S3 Time-lapse measurement of a non-invasive vulva carcinoma cell. Time series of brightfield images at the location of a non-invasive vulva carcinoma cell (A-431) embedded in a 3D collagen gel. Superimposed are contour lines encoding relative changes in strain energy density between two subsequent time steps, indicating how the distribution of strain energy density shifts around the proximity of the cell during the measurement. The cell shape is outlined in white.
(WMV)

Movie S4 Time-lapse measurement of an invasive breast carcinoma cell. Time series of brightfield images at the location of an invasive breast carcinoma cell (MDA-MD-231) embedded in a 3D collagen gel. Superimposed are contour lines encoding relative changes in strain energy density between two subsequent time steps, indicating how the distribution of strain energy density shifts around the proximity of the cell during the measurement. The cell shape is outlined in white.
(WMV)

Text S1 Detailed information about the measurement of strain energy in 3D collagen gels.
(PDF)

File S1 The MATLAB (MathWorks, Natick, MA, USA) source code of all necessary programs to carry out measurements of strain energy in 3D collagen gels.
(ZIP)
