# Supplementary figures and images for: 3D Traction Forces in Cancer Cell Invasion
Source: PLoS One. 2012 Mar 30;7(3):e33476. doi: 10.1371/journal.pone.0033476 (PMC3316584; doi:10.1371/journal.pone.0033476)

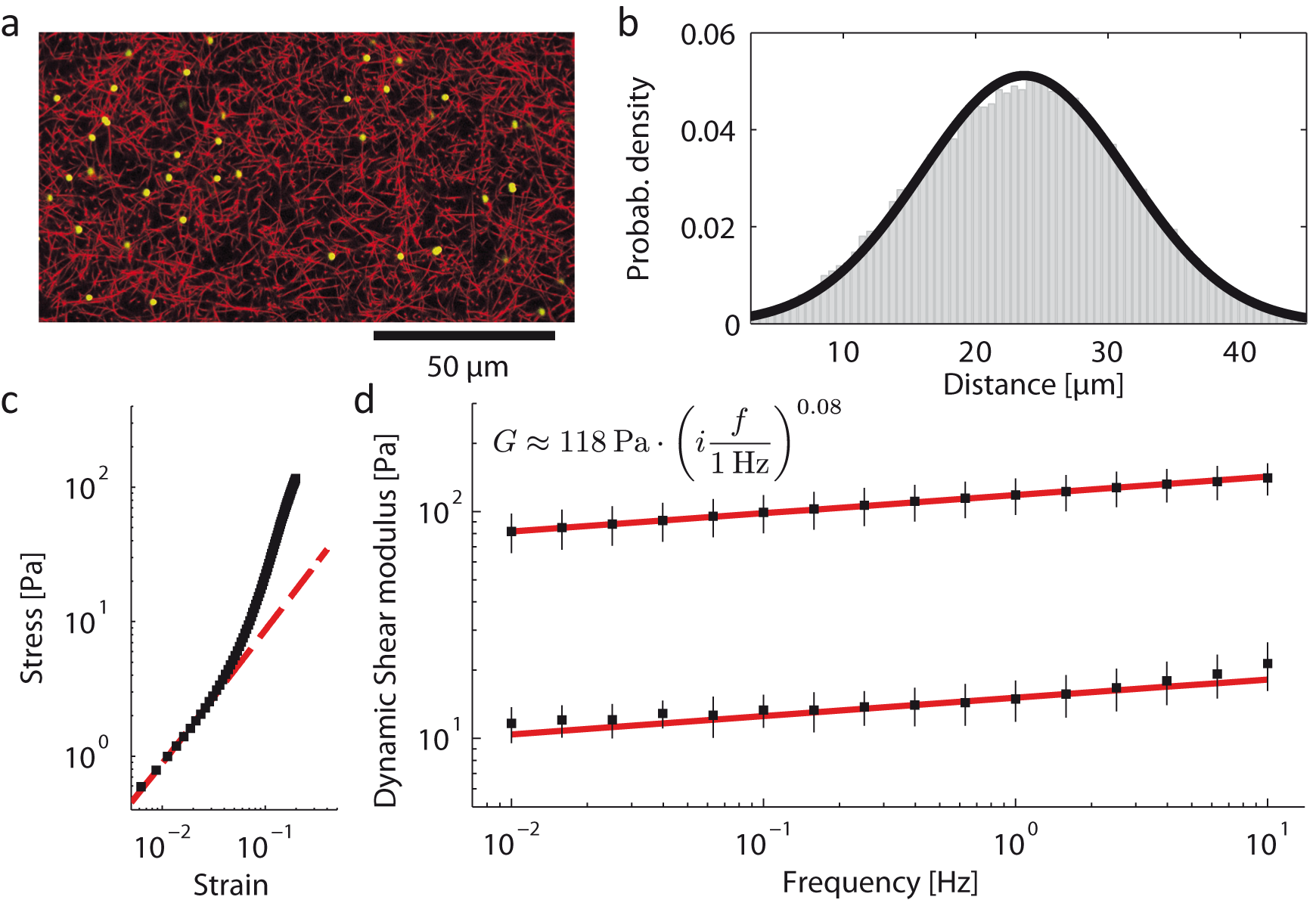

Supplement: Figure S1 — Properties of collagen gels. a) Confocal section through a collagen gel. b) Distribution of bead-bead distances. c) Stress-strain relationship of the collagen network measured in a cone-plate rheometer during a strain ramp (speed: 1%/s). The behavior is approximately linear for strains below 5%; d) Frequency response measured in a cone-plate rheometer. The amplitude of sinusoidal oscillations was 5%. The storage modulus G′ is 118 Pa at a frequency f of 1 Hz. Storage modulus G′ and loss modulus G″ increased weakly with frequency according to a power-law with exponent 0.08, indicative of predominantly elastic behavior. (TIF) [file pone.0033476.s001.tif]

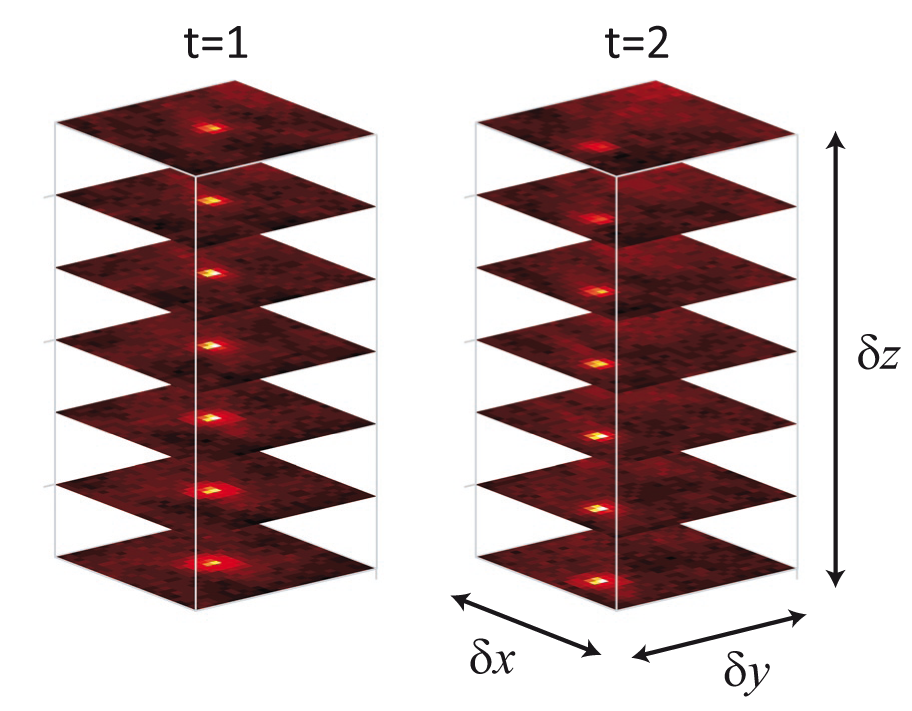

Supplement: Figure S2 — Intensity distribution in a subvolume around a bead at two distinct time points. To track the bead position, the subvolume at t = 2 is shifted until a best match with the subvolume at t = 1 is achieved. (TIF) [file pone.0033476.s002.tif]

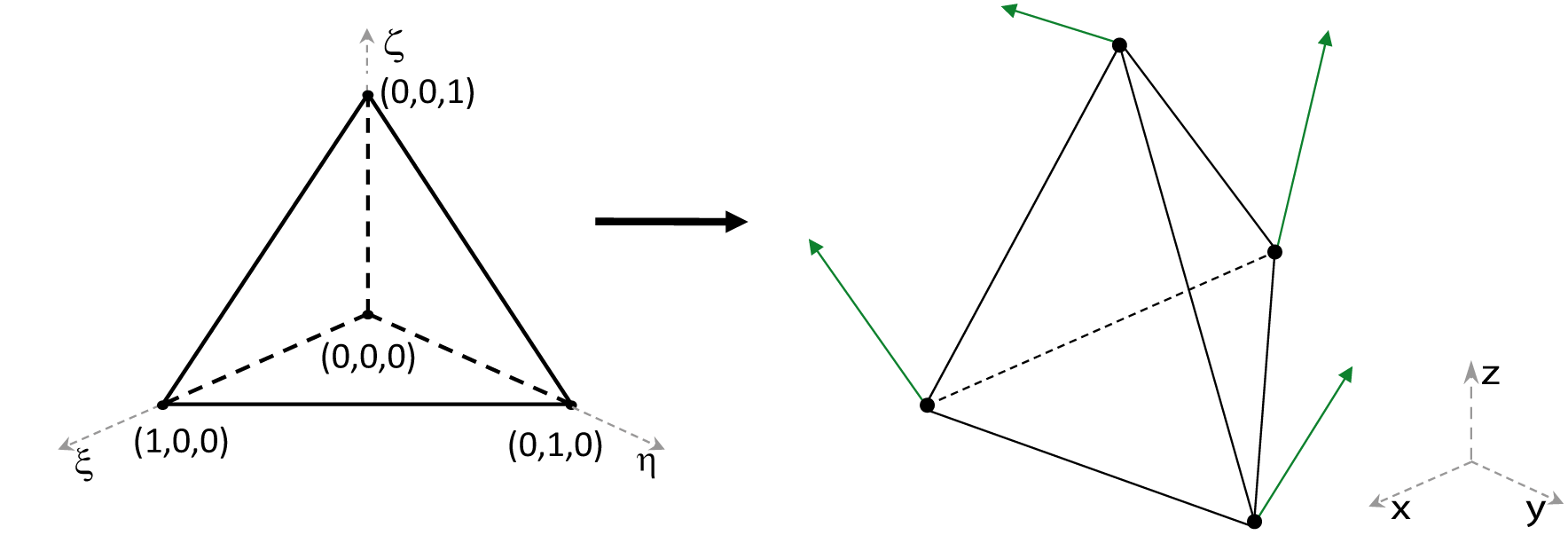

Supplement: Figure S3 — Linear interpolation of displacements. The displacements at any location within a tetrahedral element are linearly interpolated with the use of shape functions according to Eq. (5). These shape functions are defined in a fixed , , coordinate system of a parent tetrahedral element shown on the left. The map in Eq. (5) is then fully defined by the 4 corner nodes (i.e. the bead positions) of a tetrahedron in the global , , coordinate system. (TIF) [file pone.0033476.s003.tif]

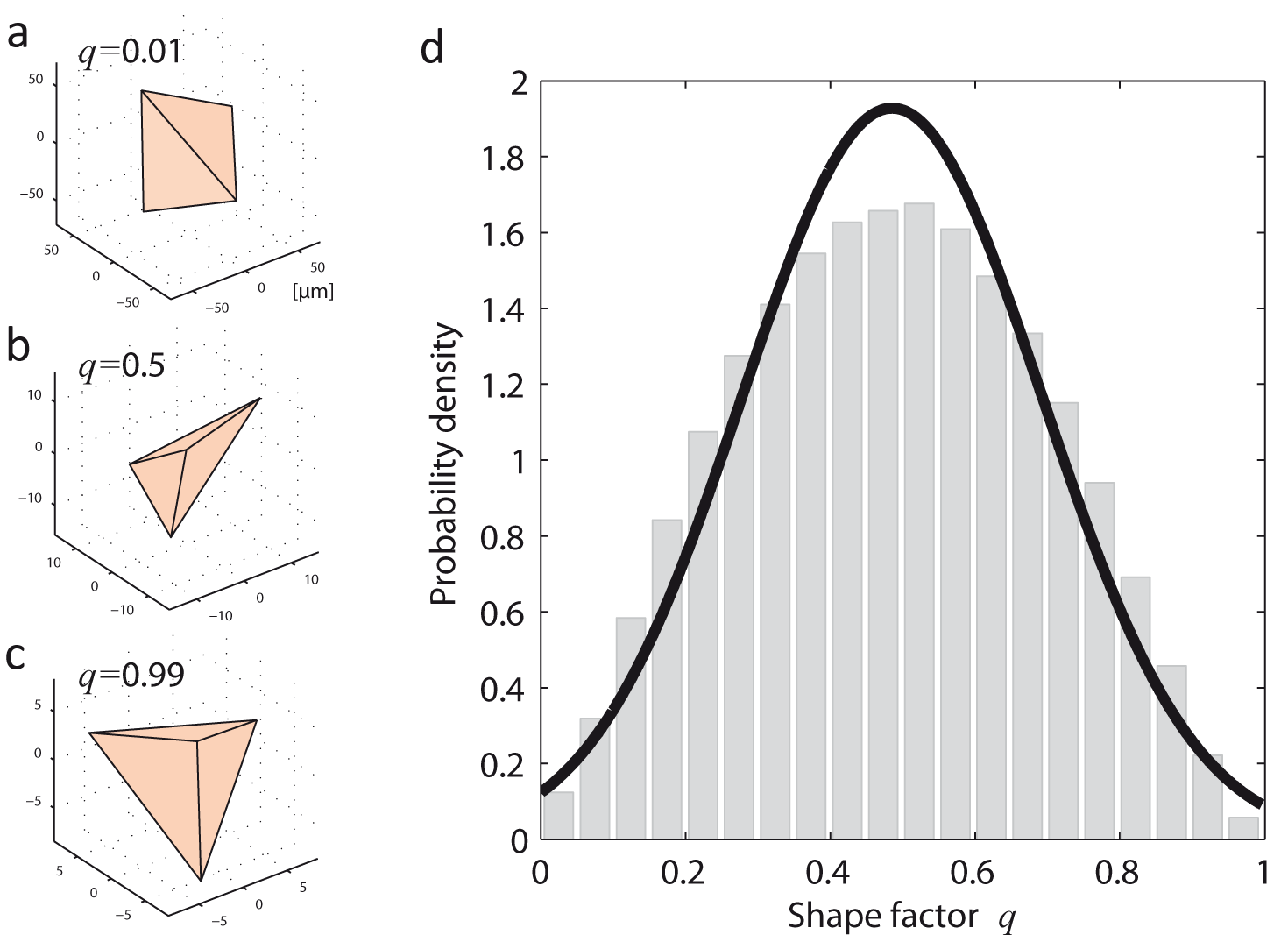

Supplement: Figure S4 — Shapes of tetrahedral elements. a–c: Tetrahedral elements of various shapes with different shape quality factors q. Elements with small shape factors have a highly degenerate geometry, e.g. they are flat (a). As the shape factors increase, the elements look increasingly like a regular tetrahedron (b and c) d: Distribution of shape quality factors in a typical collagen gel. (TIF) [file pone.0033476.s004.tif]

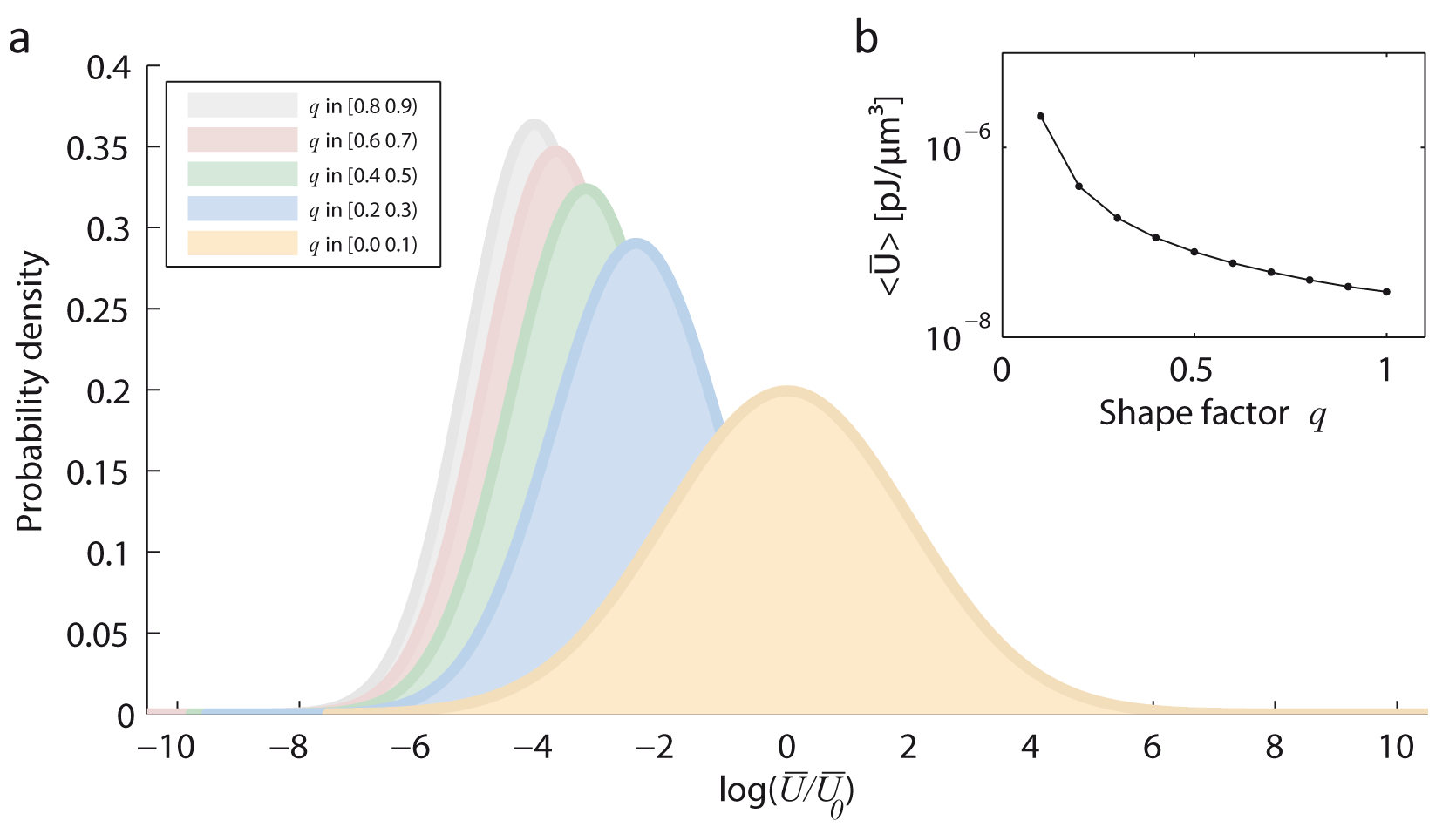

Supplement: Figure S5 — Tetrahedral shape influences erroneous strain energy density. Bead position noise causes erroneous strain energy densities ( normalized to the expected value of a tetrahedron with a shape factor of 0.1) with a log-normal probability density distribution. The expected value and the width of the distribution depend on the shape of the tetrahedra. As the shape quality factor gradually decreases, the distribution widens and the expected value increases. The inset shows the geometric mean of the erroneous strain energy as a function of the shape quality factor q. For values of q<0.5, the tetrahedral elements become excessively sensitive to noisy displacements. (TIF) [file pone.0033476.s005.tif]

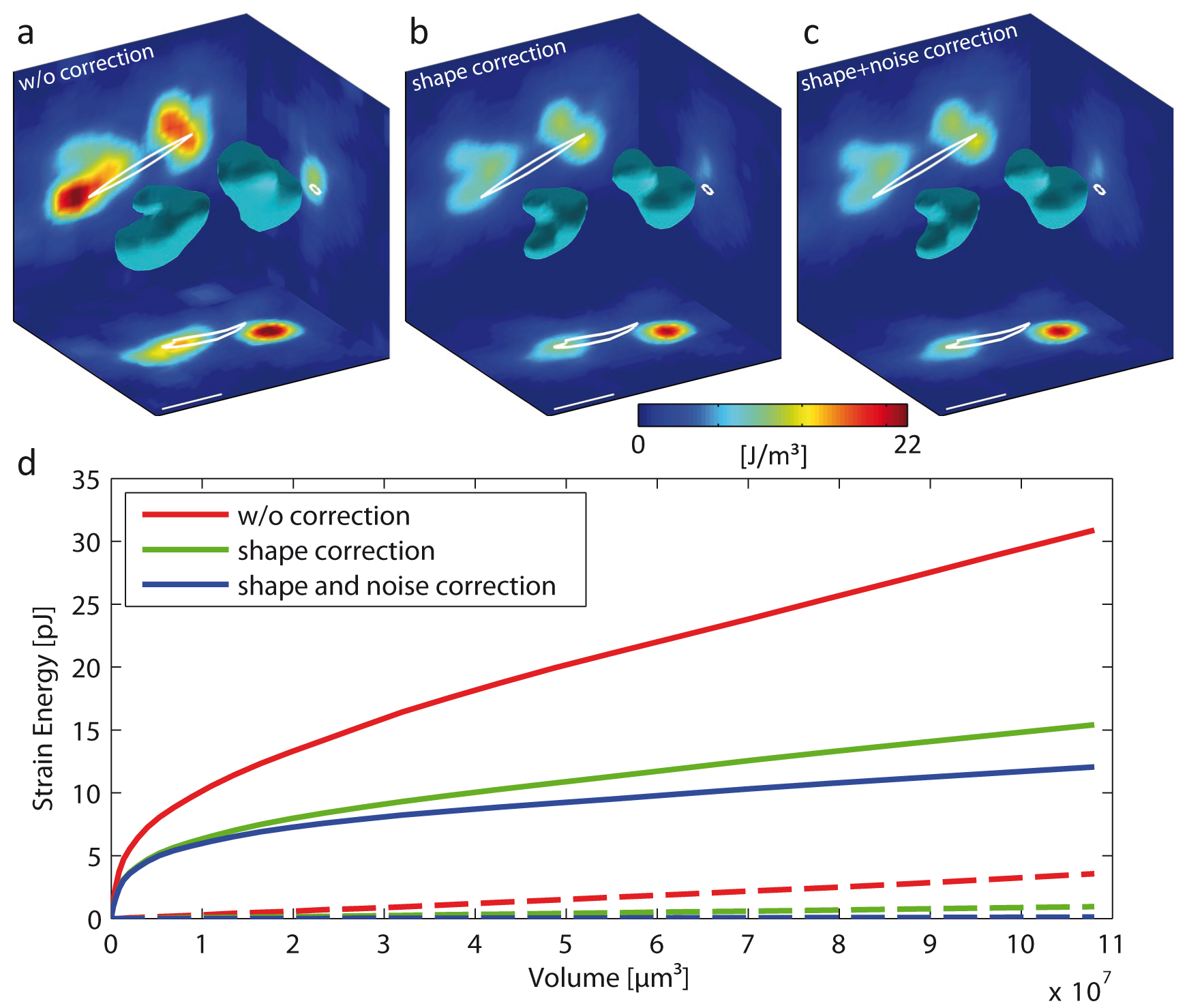

Supplement: Figure S6 — Noise correction of strain energy density. a: strain energy density distribution around an A-125 lung carcinoma cell without any correction, b: after eliminating tetrahedral elements with shape quality factor q<0.5, c: after additional noise subtraction according to Eq. (15). d: Integration of strain energy density over increasingly larger volumes shows the importance of shape and noise correction for recovering the strain energy. Here, the solid lines correspond to strain energy measurements of the cell shown in a–c; the dashed lines to the strain energy measured in a gel without cells. (TIF) [file pone.0033476.s006.tif]

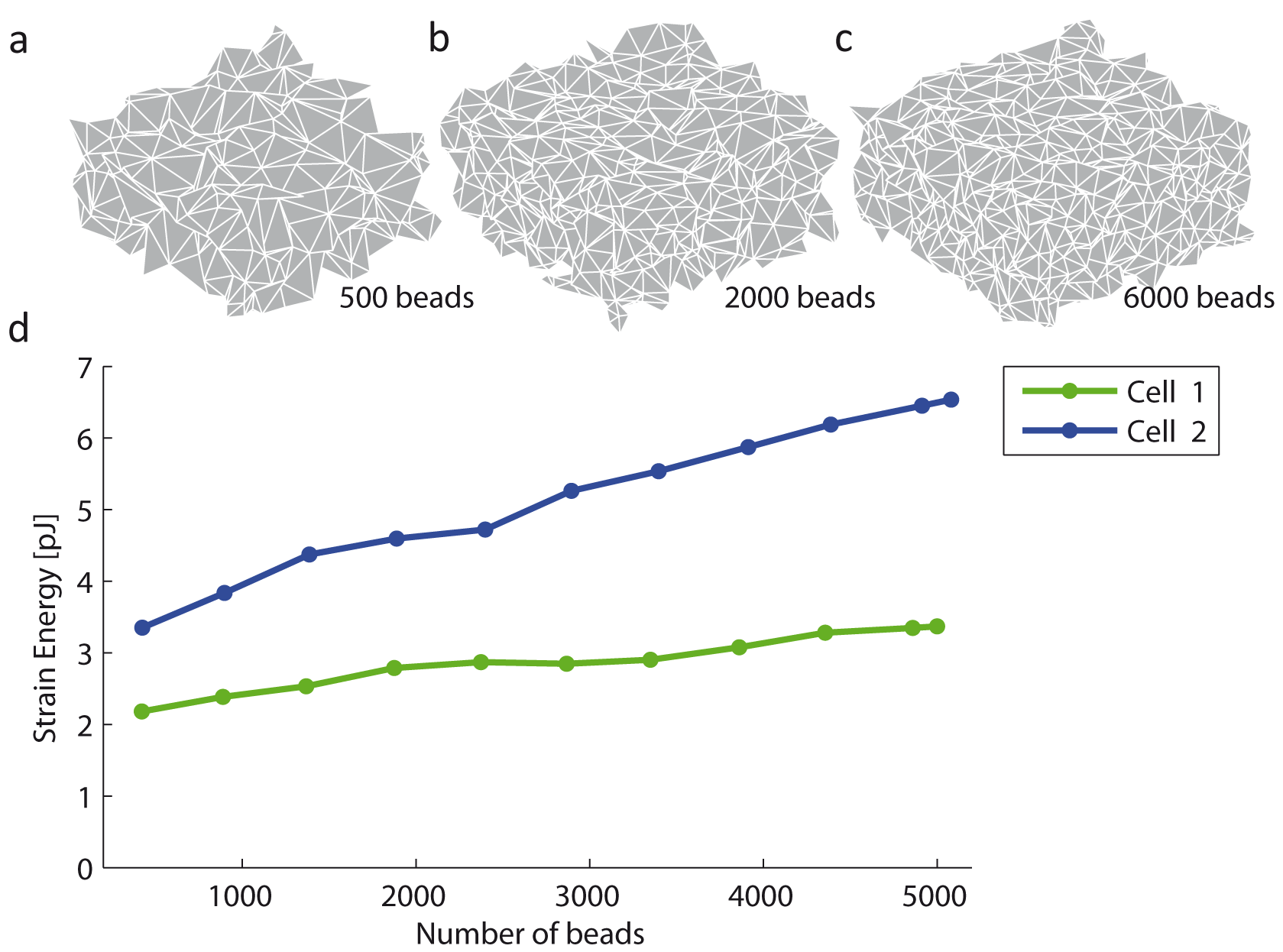

Supplement: Figure S7 — Strain energy convergence. Strain energy in a fixed volume around two representative cells evaluated on a subset of beads, i.e. the number of beads in an experimental data set was systematically reduced (a, b, and c show resulting finite element meshes) to obtain the curves in d. (TIF) [file pone.0033476.s007.tif]

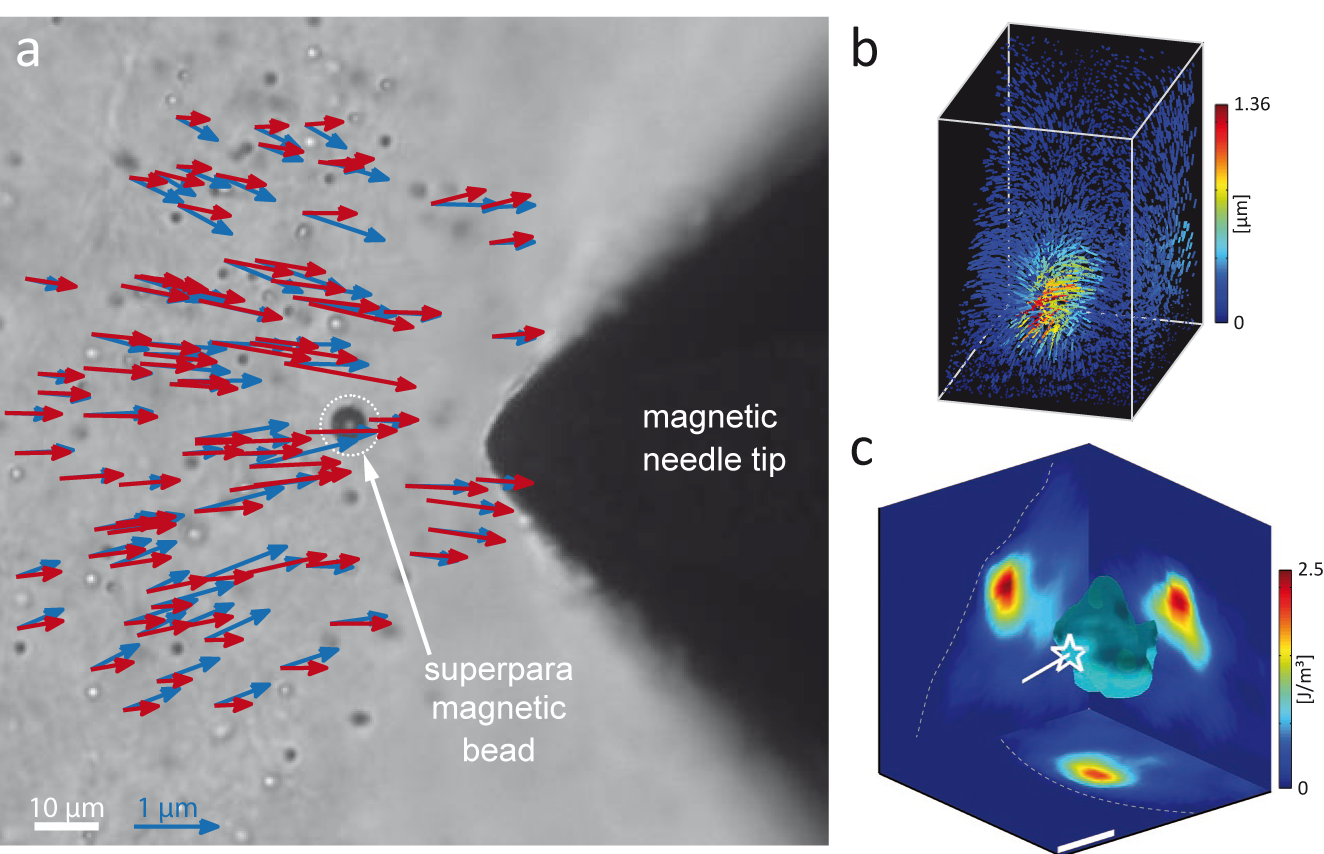

Supplement: Figure S8 — Displacements and strain energy density around a deflected superparamagnetic bead. a: Measured (blue) and fitted (red) projected displacements of fluorescent beads embedded in a collagen gel in the vicinity of a superparamagnetic bead. The gel was deformed with a force of 58 nN acting on the magnetic bead. b: Measured displacements of the fluorescent beads in the collagen drop between the force-on and force-off states. c: Corresponding strain energy density distribution around the magnetic bead indicated by the white star. The direction of the applied force is shown by the white line. (TIF) [file pone.0033476.s008.tif]

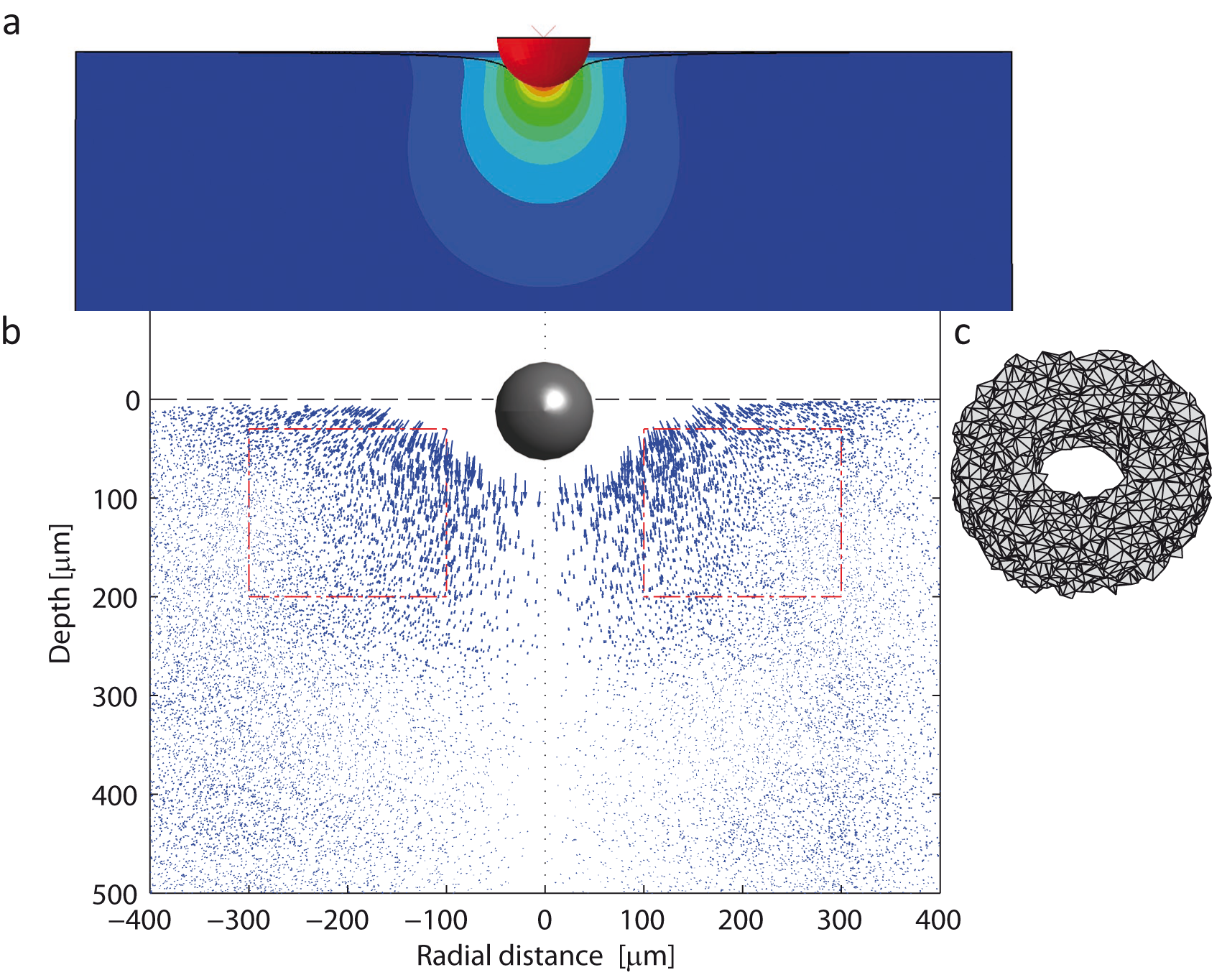

Supplement: Figure S9 — Experimental verification of the strain energy measurement by an indentation experiment with a steel ball. a: Finite Element simulation of the experiment. b: Measured displacements projected on a cylindrical coordinate system with the z axis going through the center of the steel ball. c: The strain energy was evaluated in a donut shaped subvolume as indicated by the red lines in b). (TIF) [file pone.0033476.s009.tif]

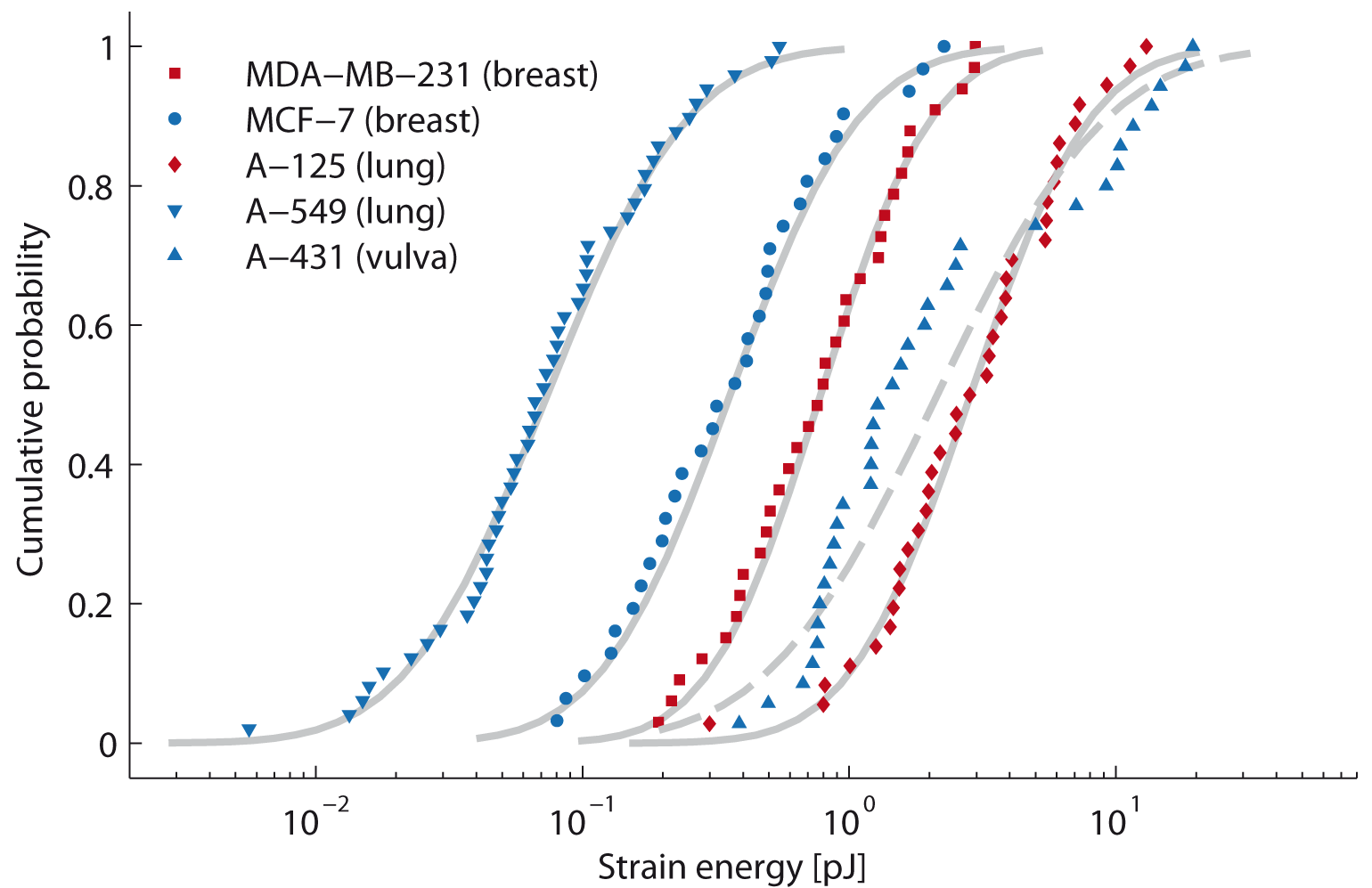

Supplement: Figure S10 — Cumulative probabilities of total strain energy for various carcinoma cell lines. Each data point corresponds to a measurement from a different cell. The lines are cumulative probabilities from a best-fit log-normal distribution. With the exception of A-431 cells, cellular strain energy data follow a log-normal distribution. (TIF) [file pone.0033476.s010.tif]

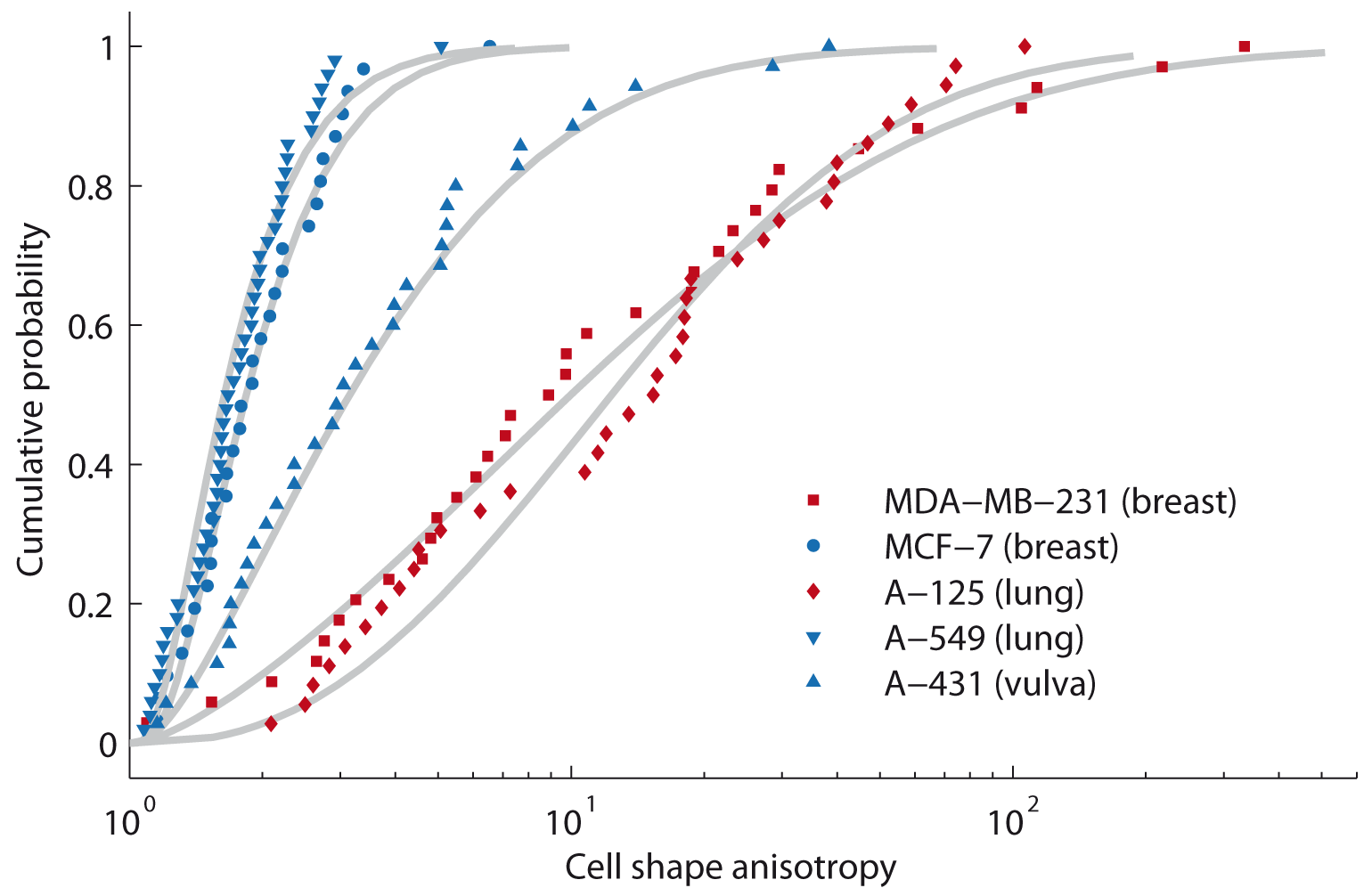

Supplement: Figure S11 — Cumulative probabilities of cell shape anisotropy for various carcinoma cell lines. Each data point corresponds to a measurement from a different cell. The lines are cumulative probabilities from a best-fit log-normal distribution. (TIF) [file pone.0033476.s011.tif]

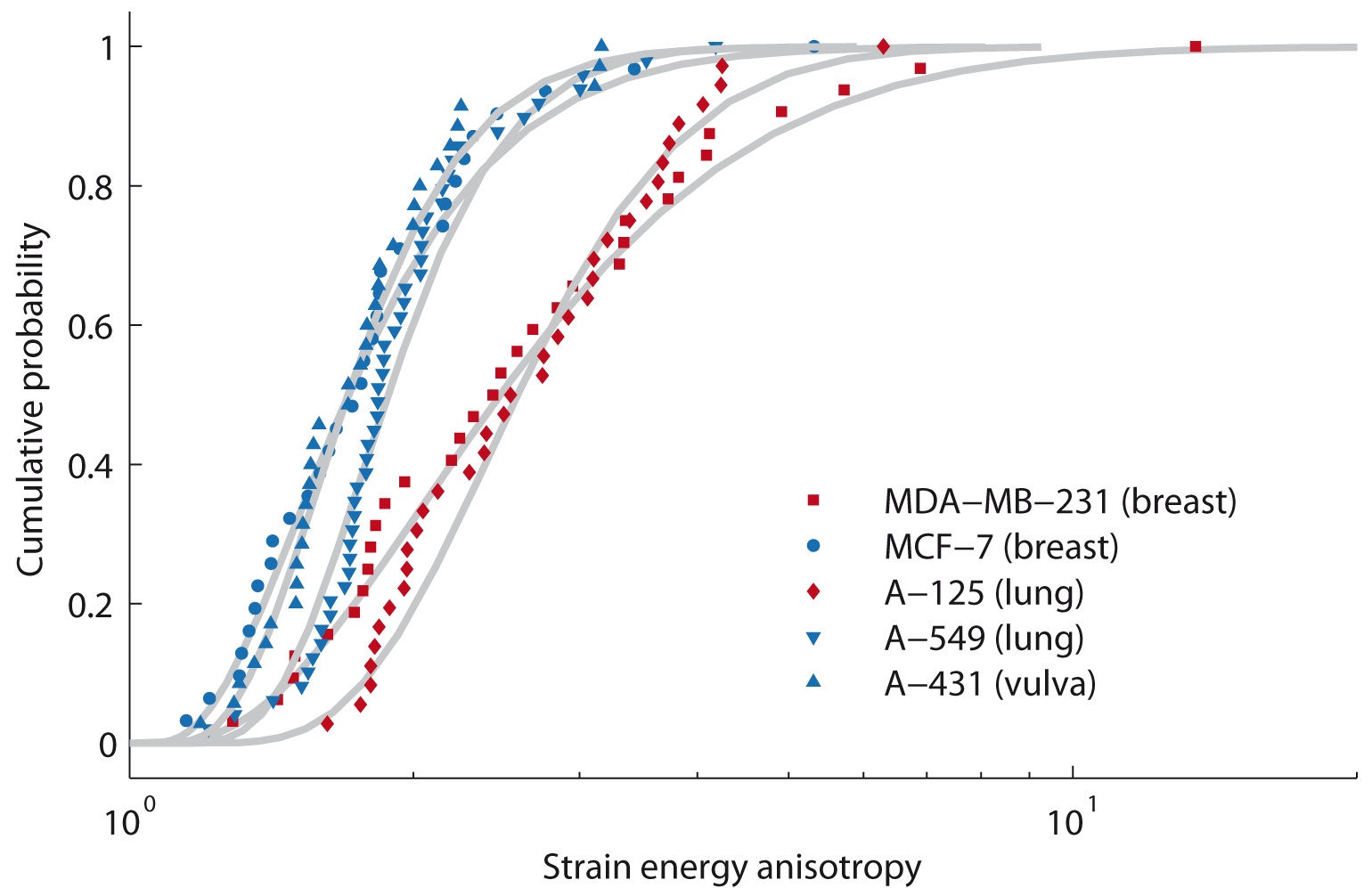

Supplement: Figure S12 — Cumulative probabilities of strain energy density anisotropy for various carcinoma cell lines. Each data point corresponds to a measurement from a different cell. The lines are cumulative probabilities from a best-fit log-normal distribution. (TIF) [file pone.0033476.s012.tif]

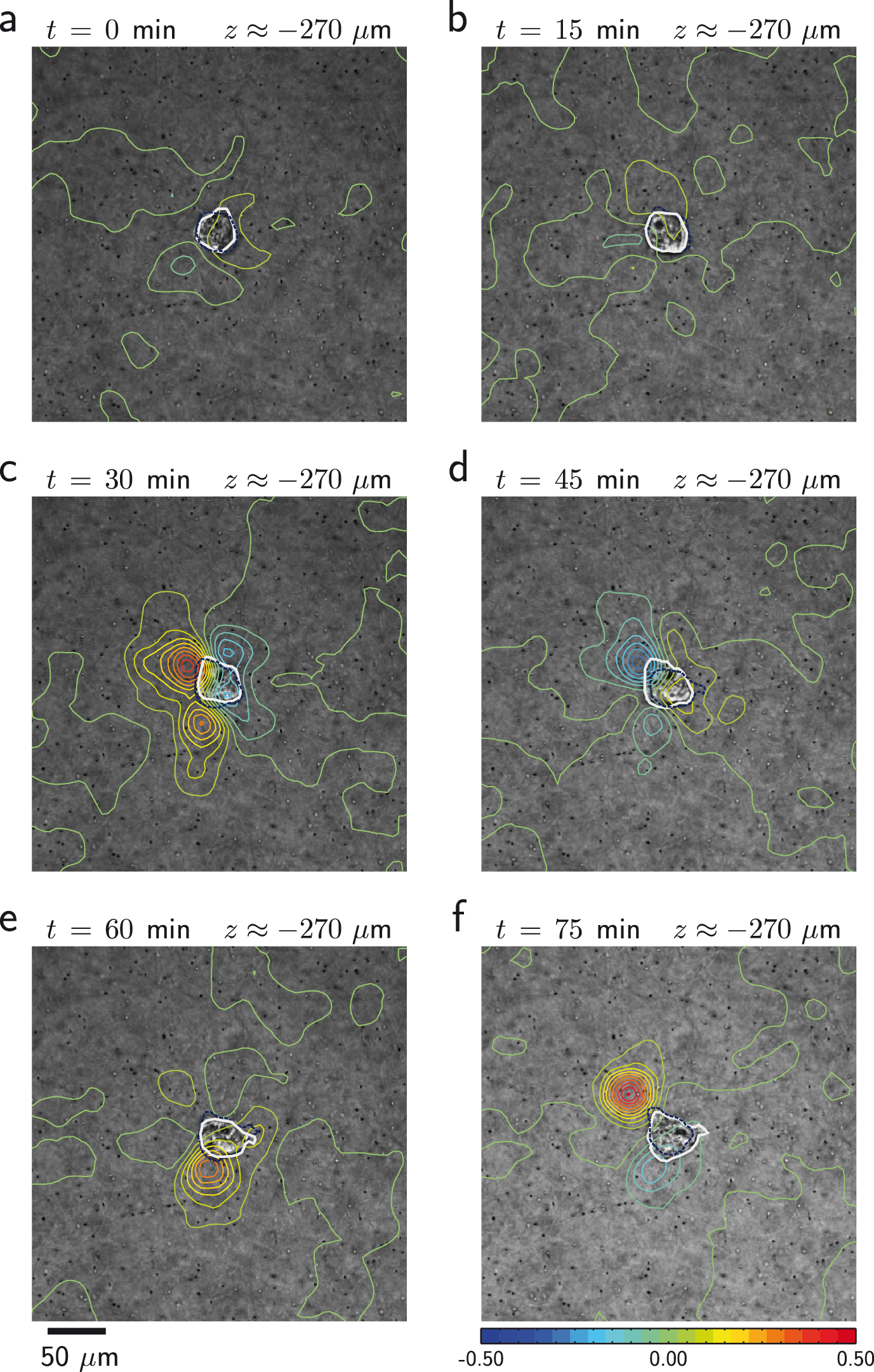

Supplement: Figure S13 — Time-lapse measurement of a non-invasive vulva carcinoma cell. Time series of brightfield images at the location of a non-invasive vulva carcinoma cell (A-431) embedded in a 3D collagen gel. Superimposed are contour lines encoding relative changes in strain energy density between two subsequent time steps, indicating how the distribution of strain energy density shifts around the proximity of the cell during the measurement. The cell shapes at the first and second time step are outlined in white and dark blue, respectively, to indicate the changes in cell position. (TIF) [file pone.0033476.s013.tif]

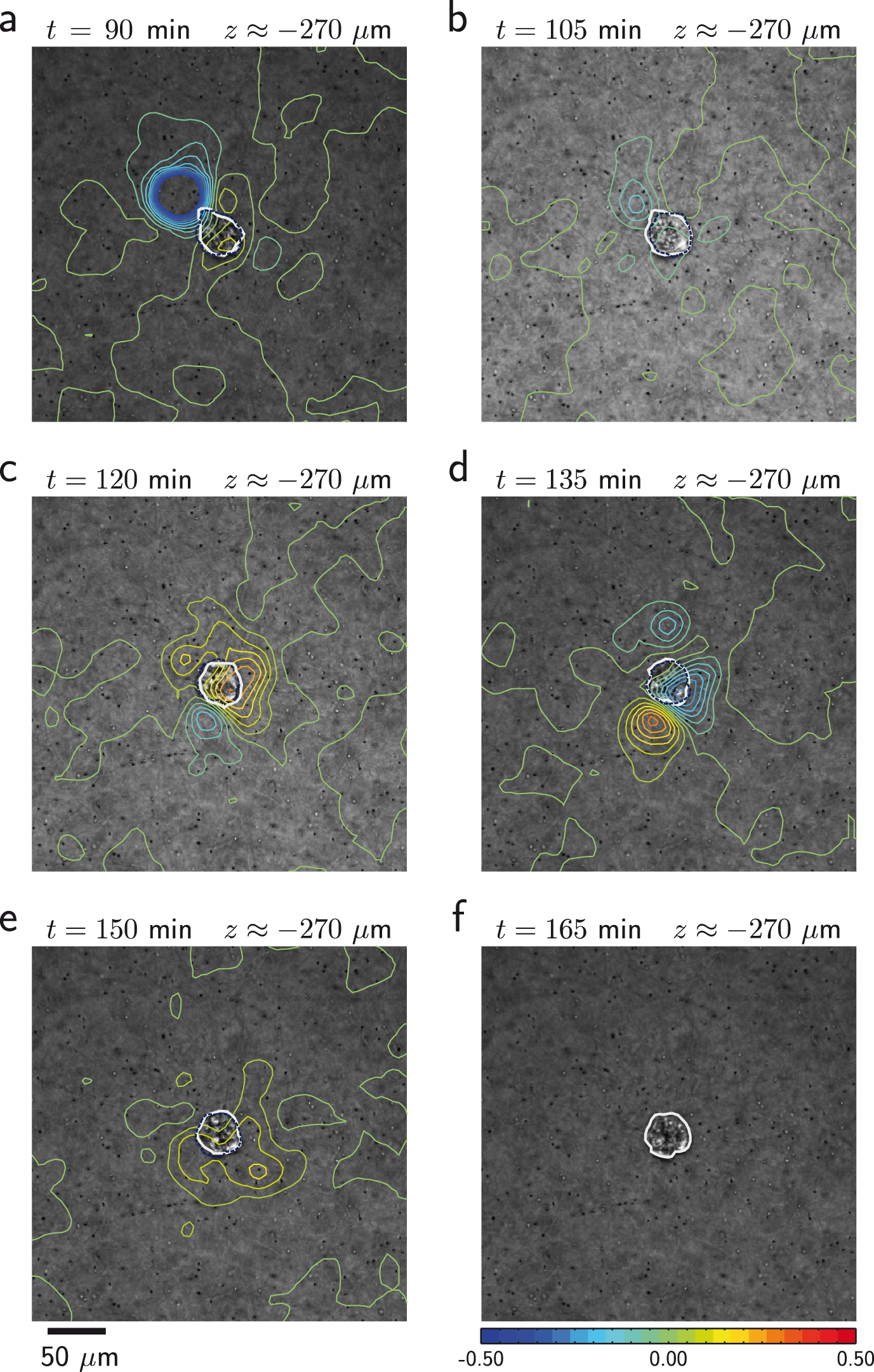

Supplement: Figure S14 — Continuation of Fig. S13. (TIF) [file pone.0033476.s014.tif]

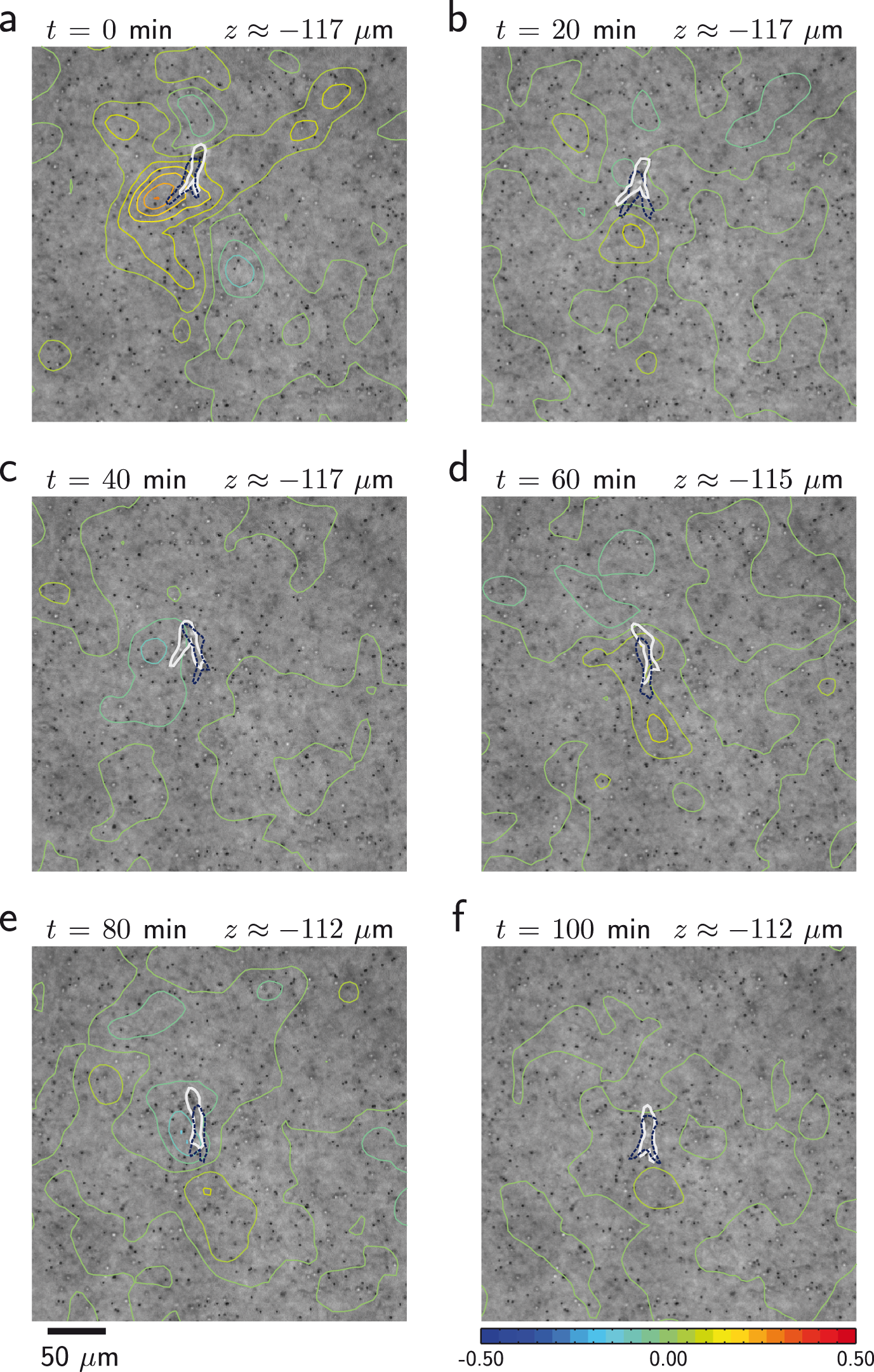

Supplement: Figure S15 — Time-lapse measurement of an invasive breast carcinoma cell. Time series of brightfield images at the location of an invasive breast carcinoma cell (MDA-MD-231) embedded in a 3D collagen gel. Superimposed are contour lines encoding relative changes in strain energy density between two subsequent time steps, indicating how the distribution of strain energy density shifts around the proximity of the cell during invasion. The cell shapes at the first and second time step are outlined in white and dark blue, respectively, to indicate the changes in cell position. (TIF) [file pone.0033476.s015.tif]

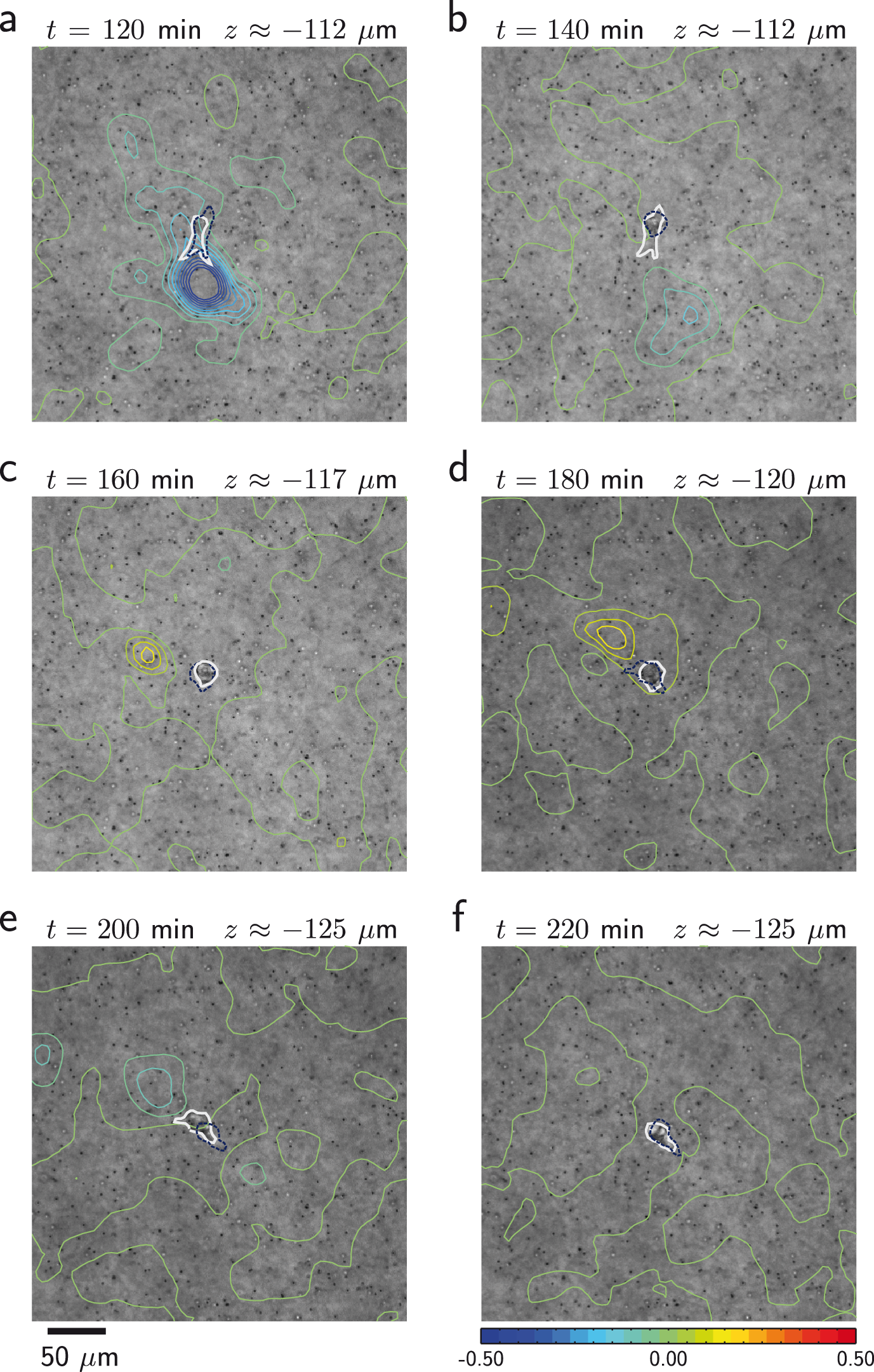

Supplement: Figure S16 — Continuation of Fig. S15. (TIF) [file pone.0033476.s016.tif]

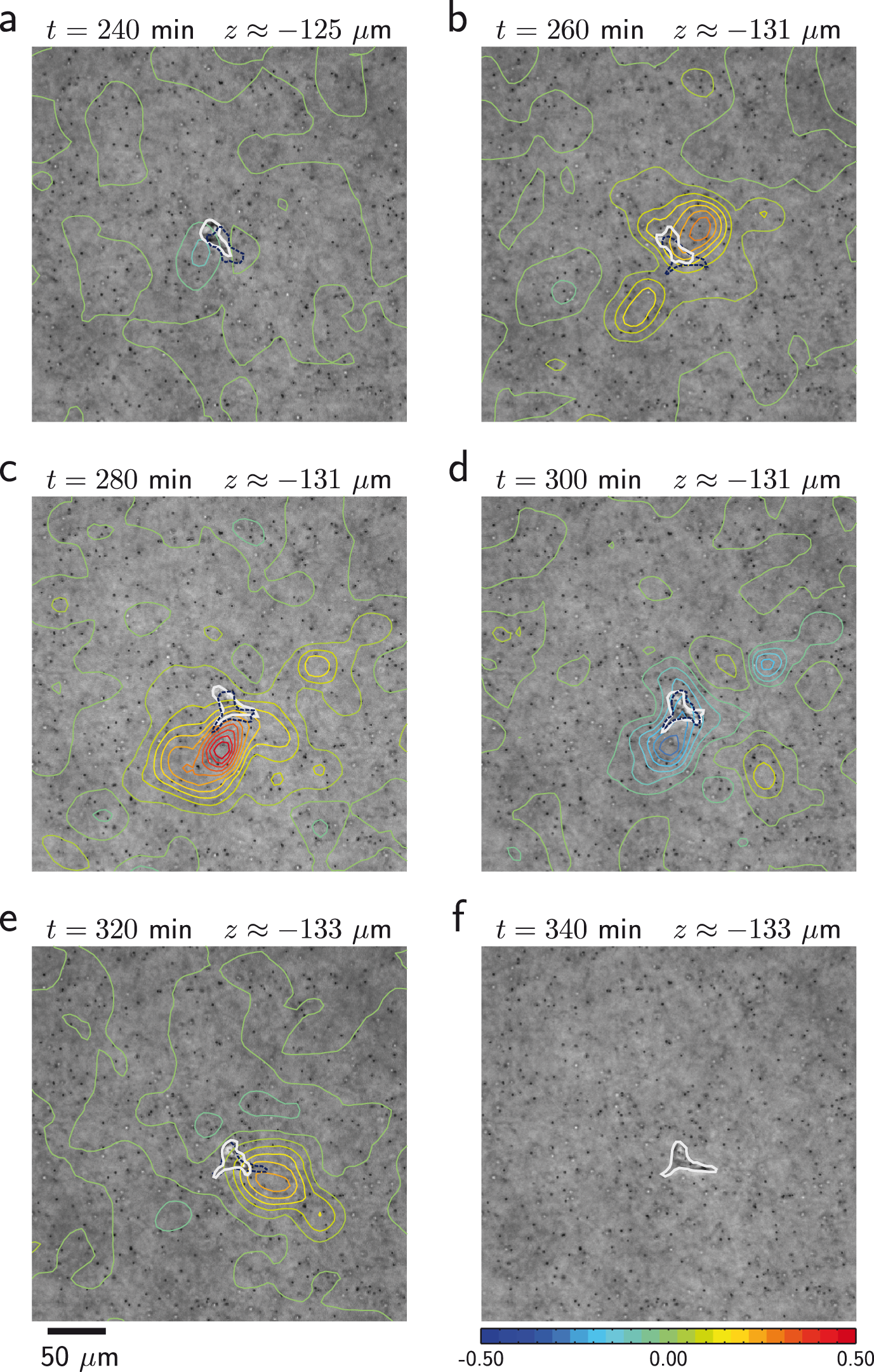

Supplement: Figure S17 — Continuation of Fig. S16. (TIF) [file pone.0033476.s017.tif]
