# Supplementary material for: 3D Traction Forces in Cancer Cell Invasion
Source: PLoS One. 2012 Mar 30;7(3):e33476. doi: 10.1371/journal.pone.0033476 (PMC3316584; doi:10.1371/journal.pone.0033476)
Supplement: Text S1 — Detailed information about the measurement of strain energy in 3D collagen gels. (PDF) [file pone.0033476.s022.pdf]

Supporting Information

Measurement of strain energy in 3D collagen gels

Principle

Cells migrating through a 3D extracellular matrix (ECM) exert forces on their surroundings and thereby deform the matrix. The mechanical work performed by the cells during migration, specifically the mechanical work that is imparted by the cell to deform the matrix, is temporarily stored by the elastic matrix fibers in the form of an elastic strain energy. If the mechanical properties of the matrix are known, the strain energy stored in the matrix can be determined from a measurement of the matrix deformation. There are three main steps involved:

1. Characterizing the mechanical properties of the ECM;
2. Measuring the cell-induced deformation in the ECM;
3. Computing the strain energy stored in the deformed ECM.

The following sections give details for each step.

Mechanical properties of collagen gels

Reconstituted collagen gels show a fibrous networks structure (Fig. S1a). At the collagen concentration used here (2.4 mg/ml collagen), the average pore size of the network is 1.3 µm[1]. Fluorescent microbeads of 1 µm diameter were dispersed in the gels prior to collagen polymerization and served as fiducial markers. These beads are trapped in the gel by tightly binding to collagen fibers. The average bead-bead distance is (24 ± 8) µm (Fig. S1b). At this length scale, we can neglect the porous or fibrous microstructure of the collagen and treat the collagen gels as a continuum with isotropic properties[1].

The elastic properties of the collagen gels were measured with a rate-controlled cone-plate rheometer (AR-G2, TA Instruments, New Castle, DE, USA) by applying oscillatory shear strain with an amplitude of 5%. Gels were prepared as described in Methods but allowed to polymerize *in situ* (between the rheometer plates) to ensure good contact with the probing cone-plate. Measurements were carried out at a temperature of 37°C. The frequency response of the complex modulus shows predominantly elastic behavior at all frequencies measured (Fig. S1d). Collagen gels display a non-linear stress-strain relationship (Fig. S1c) with prominent strain stiffening at strains above 5%. The method used to extract the strain energy described below ignores non-linear behavior and treats the collagen gels as isotropic linear elastic materials with a shear modulus of G = 118 Pa.

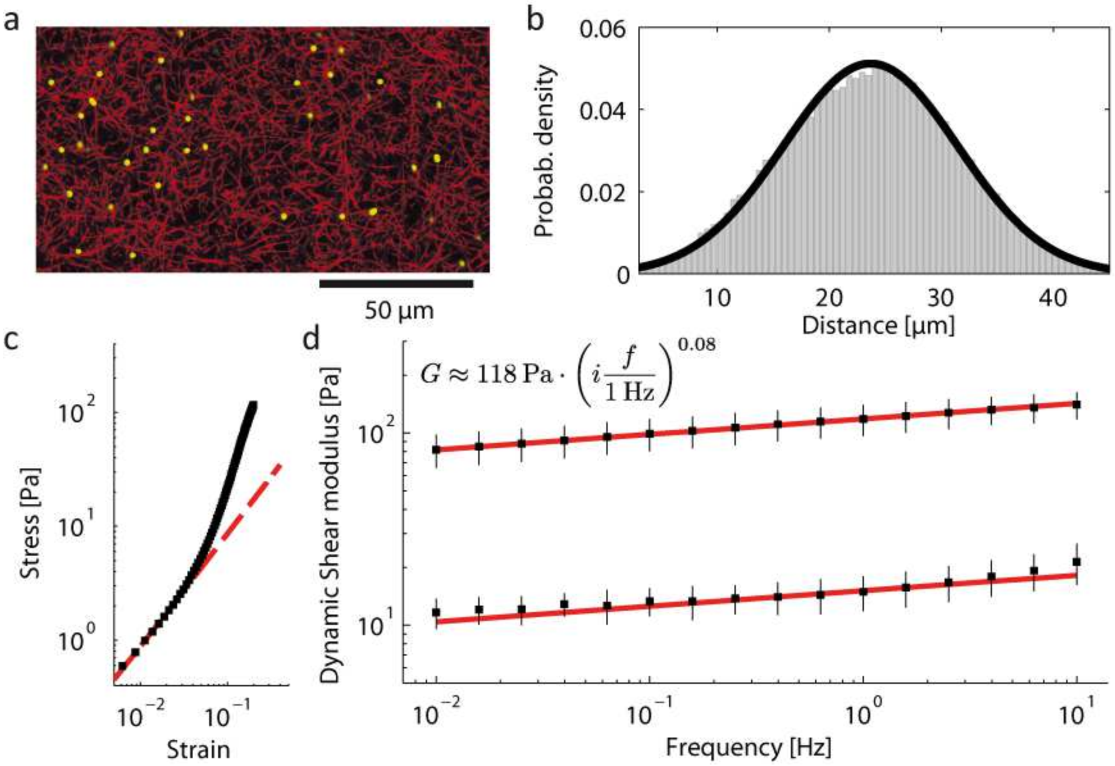

Figure S1: Properties of collagen gels. a) Confocal section through a collagen gel. b) Distribution of bead-bead distances. c) Stress-strain relationship of the collagen network measured in a cone-plate rheometer during a strain ramp (speed: 1%/s). The behavior is approximately linear for strains below 5%; d) Frequency response measured in a cone-plate rheometer. The amplitude of sinusoidal oscillations was 5%. The storage modulus G' is 118 Pa at a frequency f of 1 Hz. Storage modulus G' and loss modulus G" increased weakly with frequency according to a power-law with exponent 0.08, indicative of predominantly elastic behavior.

Measurement of the displacement field

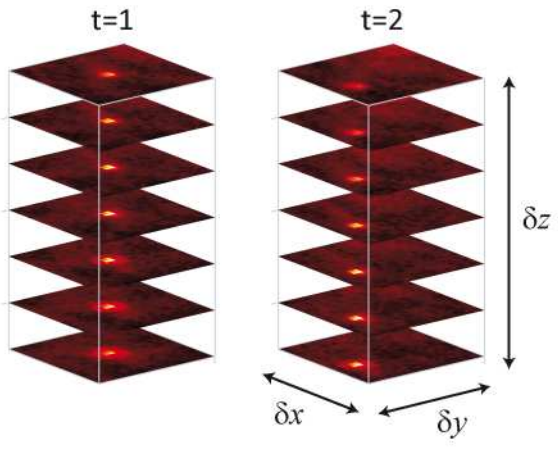

Figure S2: Intensity distribution in a subvolume around a bead at two distinct time points. To track the bead position, the subvolume at t=2 is shifted until a best match with the subvolume at t=1 is achieved.

Matrix displacments are inferred from measurements of the 3D positions of fluorescent beads that are scattered throughout the gel. Optical sections (z-stacks) are taken throughout the depth of the gel at 2 µm intervals at two or more time points. The 3D positions of the beads and their displacements over time can then be determined as follows: In a first step, the positions of the beads during the first time point are determined from the positions of all pixels in the image stack with a local intensity maximum above a specific intensity threshold. Second, all pixels in a 7x7x7 pixel

neighborhood ((4.5 x 4.5 x 14) µm) around these local maximum pixels are selected. The displacement vector of each bead is determined using a 3D difference-with-interpolation algorithm that shifts the 7x7x7 neighborhood pixels around each bead from the second image stack by sub-pixel increments in the three spatial directions $\delta x, \delta y, \delta z$. Let p_{xyz} be the pixel intensities from the first image stack, and let $c_{xyz}(\delta x, \delta y, \delta z)$ be the intensities of the pixels in the shifted second stack, obtained by tri-linear interpolation on $c_{xyz}(0,0,0)$, with the coordinate origin at the center of the 7x7x7 pixel neighborhood. The shift $(\delta x, \delta y, \delta z)$ is determined by minimizing the squared differences between p_{xyz} and c_{xyz}:

$$(\delta x, \delta y, \delta z) = \arg\min \sum_{x,y,z} \left(c_{xyz}(\delta x, \delta y, \delta z) - p_{xyz}\right)^2 \tag{1}$$

This procedure yields subpixel resolution. The accuracy of the method was estimated by repeatedly measuring bead positions in a cell-free collagen gel and found to be 22 nm in the x- and y-direction, and 130 nm in the z-direction (20x 0.4 NA objective, 0.5x optical coupler, z-section distance of 2 µm).

Calculation of strain energy

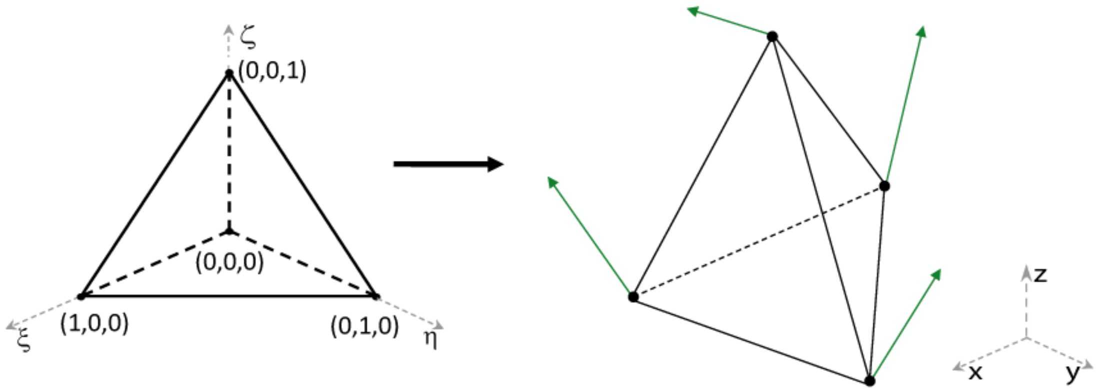

Figure S3: Linear interpolation of displacements. The displacements at any location within a tetrahedral element are linearly interpolated with the use of shape functions according to Eq. (5). These shape functions are defined in a fixed ξ, η, ζ coordinate system of a parent tetrahedral element shown on the left. The map in Eq. (5) is then fully defined by the 4 corner nodes (i.e. the bead positions) of a tetrahedron in the global x, y, z coordinate system.

This section outlines the computation of the strain energy. More details can be found elsewhere[2].

In a continuum material, the elastic strain $\boldsymbol{\varepsilon}$ at any spatial position can be calculated from the displacements **u** according to

$$\boldsymbol{\varepsilon} = \frac{1}{2}\left((\nabla \mathbf{u})^T + (\nabla \mathbf{u})\right). \tag{2}$$

If we assume isotropic linear elastic properties, the stress $\boldsymbol{\sigma}$ is related to strain $\boldsymbol{\varepsilon}$ by Hook's law

$$\boldsymbol{\sigma} = \mathbf{D} \cdot \boldsymbol{\varepsilon}, \tag{3}$$

where $\mathbf{D}$ is the material stiffness matrix. $\mathbf{D}$ is fully defined by two material constants, e.g. Young's modulus and Poisson's ratio. The strain energy U due to elastic deformation is then the volume integral of the inner product of stress and strain, which according to Eq. (3) takes the form

$$U = \frac{1}{2}\int \boldsymbol{\varepsilon}^T \cdot \boldsymbol{\sigma}\, dV = \frac{1}{2}\int \boldsymbol{\varepsilon}^T \cdot \mathbf{D} \cdot \boldsymbol{\varepsilon}\, dV \tag{4}$$

To solve the above integral, the deformation of the material everywhere in the measurement volume needs to be known. However, we only know the material displacements at the discrete locations of the beads. Displacements between bead positions are therefore obtained by tri-linear interpolation through a Delaunay tessellation[3] with linear tetrahedral finite elements. Thus, four bead positions serve as nodes for a tetrahedral element as illustrated in Fig. S3. For convenience, we write the 12 displacement components (4 nodes with 3 displacement components in x-, y-, and z- direction) as a vector $\mathbf{u}_b$. The interpolation can then be written as a product with suitable shape functions $\mathbf{N}$

$$\mathbf{u} = \mathbf{N} \cdot \mathbf{u}_b \tag{5}$$

Similarly, we write the 6 components of the symmetric strain tensor $\boldsymbol{\varepsilon}$ as a vector in the form

$$\boldsymbol{\varepsilon} = \begin{pmatrix}\varepsilon_{11} & \varepsilon_{22} & \varepsilon_{33} & 2\varepsilon_{23} & 2\varepsilon_{13} & 2\varepsilon_{12}\end{pmatrix}^T \tag{6}$$

These strain components are then calculated from the displacements in Eq. (5) as

$$\boldsymbol{\varepsilon} = \mathbf{L} \cdot \mathbf{u} \tag{7}$$

where $\mathbf{L}$ is

$$\mathbf{L} = \begin{pmatrix} \frac{\partial}{\partial x} & 0 & 0 \\ 0 & \frac{\partial}{\partial y} & 0 \\ 0 & 0 & \frac{\partial}{\partial z} \\ 0 & \frac{\partial}{\partial z} & \frac{\partial}{\partial y} \\ \frac{\partial}{\partial z} & 0 & \frac{\partial}{\partial x} \\ \frac{\partial}{\partial y} & \frac{\partial}{\partial x} & 0 \end{pmatrix} \tag{8}$$

Combining Eq. (7) and Eq. (5) gives

$$\boldsymbol{\varepsilon} = \mathbf{LN} \cdot \mathbf{u}_b =: \mathbf{B} \cdot \mathbf{u}_b \tag{9}$$

with the strain-displacement matrix $\mathbf{B}$ containing the spatial derivatives of the shape functions N. Inserting Eq. (9) in Eq. (4) gives the discretized form for the strain energy

$$U = \frac{1}{2}\mathbf{u}_b^T \cdot \left(\int \mathbf{B}^T \cdot \mathbf{D} \cdot \mathbf{B}\, dV\right) \cdot \mathbf{u}_b\,, \tag{10}$$

where the volume integral defines the stiffness matrix $\mathbf{K}$ for the elements. The strain energy of one element can therefore be calculated from the measured displacements of four beads by

$$U = \frac{1}{2}\mathbf{u}_b^T \cdot \mathbf{K} \cdot \mathbf{u}_b \,. \tag{11}$$

The method outlined in this section was implemented in Matlab (MathWorks, Natick, MA, USA)[4].

Accuracy of strain energy measurements

Sources of error

A main source for error in the calculation of strain energy is the measurement noise in the bead displacements. How displacement noise is translated into erroneous strain energy is governed by two element properties, its volume and its shape, as explained below. It can be shown that the strain energy density of a linear tetrahedral element $\overline{U}$ due to given displacements (e.g. noisy bead displacements) scales inversely with the volume of the element by a power law with exponent 2/3:

$$\overline{U} = \overline{U}_0 V^{-\frac{2}{3}} \tag{12}$$

Consequently, increasing the number of beads in the measurement volume with the aim of improving the resolution of the strain energy also leads to elements with smaller volumes and therefore increased levels of measurement noise.

The optimal shape of a tetrahedron in terms of insensitivity to displacement noise is a regular tetrahedron with all six edges of equal length. After tessellation of randomly distributed beads in a given volume, however, the resulting tetrahedra can have all possible shapes including degenerate forms such as an almost flat tetrahedron. Flat tetrahedral elements are exceptionally prone to erroneous strain energy as small displacement errors can translate into large strains. To characterize the shape of tetrahedral elements, we determine the ratio of the volume of the element to the sum of its cubed edge lengths, normalized to range between 0 and 1:

$$q = \frac{36\sqrt{2} \cdot V}{\sum_{i=1}^{6} l_i^3} \tag{13}$$

Thus, a regular tetrahedron has a shape of $q=1$. The value of q decreases with increasingly degenerate forms (Fig. S4a-c). In a typical collagen gel, tetrahedra of all shapes can be found, with shape factors around 0.5 being the most frequent (Fig. S4d).

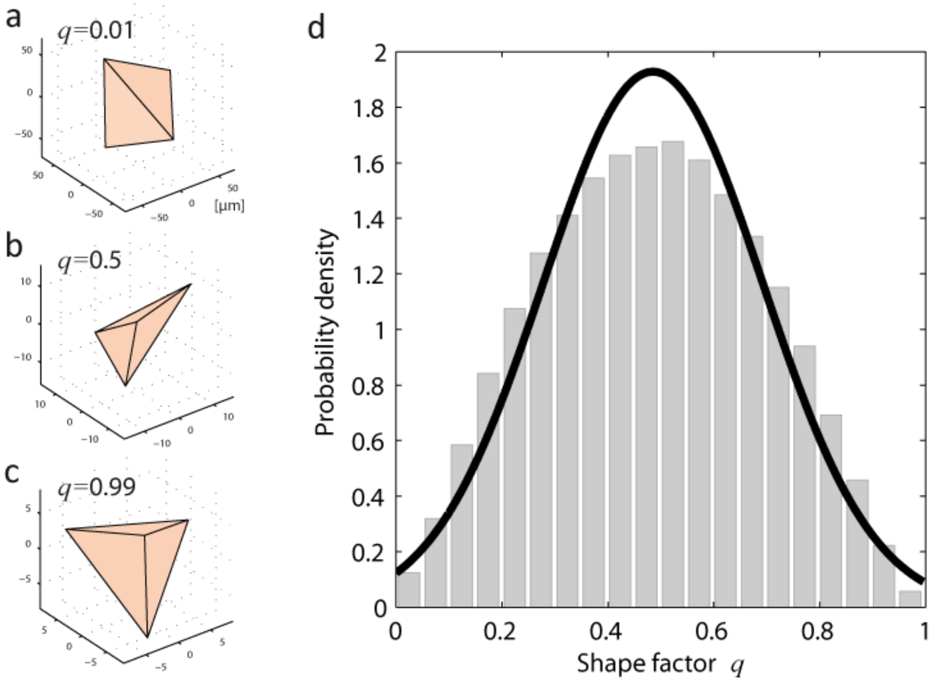

Figure S4: Shapes of tetrahedral elements. a-c: Tetrahedral elements of various shapes with different shape quality factors q. Elements with small shape factors have a highly degenerate geometry, e.g. they are flat (a). As the shape factors increase, the elements look increasingly like a regular tetrahedron (b and c) d: Distribution of shape quality factors in a typical collagen gel.

The dependence of erroneous strain energy on the shape factor was estimated from the measured positions of ~40,000 beads in a typical collagen gel, superimposed by random Gaussian displacement noise according to our experimentally determined values of 22 nm (x,y-direction) and 130 nm (z-direction). We find that Gaussian displacement noise causes an erroneous strain energy density with approximately log-normal distribution (Fig. S5a). The width of the distribution and the expected value of the erroneous strain energy increase dramatically as q falls below 0.5 (Fig. S5a-b).

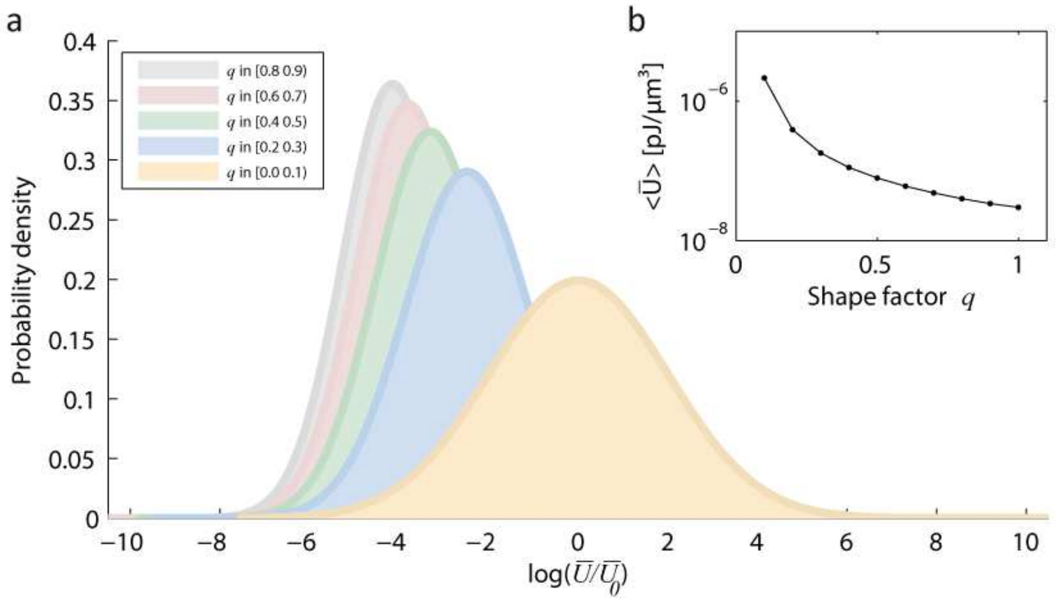

Figure S5: Tetrahedral shape influences erroneous strain energy density. Bead position noise causes erroneous strain energy densities ($\overline{U}$ normalized to the expected value $\overline{U}_0$ of a tetrahedron with a shape factor of 0.1) with a log-normal probability density distribution. The expected value and the width of the distribution depend on the shape of the tetrahedra. As the shape quality factor gradually decreases, the distribution widens and the expected value increases. The inset shows the geometric mean of the erroneous strain energy as a function of the shape quality factor q. For values of q<0.5, the tetrahedral elements become excessively sensitive to noisy displacements.

Error correction for individual elements

In the following, we describe how the erroneous strain energy from measurement noise contributes to the total strain energy, and how this error can be corrected for.

The measured displacements $\mathbf{u}_m$ are normally distributed with mean $\boldsymbol{\mu}$ and standard deviations (rms measurement noise) of σ_x, σ_y and σ_z in the x-, y-, and z-direction, respectively. The expected value of the strain energy $\langle U(\mathbf{u}_m)\rangle$ is the sum of the true strain energy and a term ΔU that represents the erroneous strain energy due to measurement noise in the bead positions and that is independent of the mean displacements.

$$U = \langle U(\mathbf{u}_m)\rangle = \frac{1}{2}\boldsymbol{\mu}^T \cdot \mathbf{K} \cdot \boldsymbol{\mu} + \Delta U \tag{14}$$

ΔU is given by

$$\Delta U = \frac{1}{2}\left(\sum_{i=1}^{12} K_{ii}\sigma_i^2\right) \tag{15}$$

where

$$\sigma_i = \begin{cases} \sigma_x \text{ for } i \in \{1,4,7,10\} \\ \sigma_y \text{ for } i \in \{2,5,8,11\} \\ \sigma_z \text{ for } i \in \{3,6,9,12\} \end{cases} \tag{16}$$

ΔU can be subtracted from the measured strain energy (Eq. (11)) of each element.

Strain energy error correction

The following strategy was employed to correct for errors in the total strain energy:

1. Elements with a shape factor of $q < 0.5$ are discarded.
2. A strain energy error evaluated with Eq. (15) and Eq. (16) is subtracted from the strain energy values of an element.
3. Gaps in the strain energy density distribution resulting from step 1 above are filled by nearest neighbor interpolation.

The impact of this error correction on the strain energy density distribution around a cell is illustrated in Fig. S6. The strain energy density distribution is plotted for each of the various correction steps described above. While the strain energy density seems little affected by the correction steps, the importance of error correction becomes evident when the total strain energy is computed by integration of the strain energy density over increasingly larger volumes, starting from the cell center (Fig. S6d). In all cases, i.e. without or with error correction, the strain energy rises rapidly at first, followed by an increase with smaller slope that is sensitive to noise. Because normally-distributed displacement noise results in a log-normal distribution of the erroneous strain energy in tetrahedral elements with similar shape factors (Fig. S5), the error subtraction approach (Eqs. (15) and (16)) is necessarily incomplete.

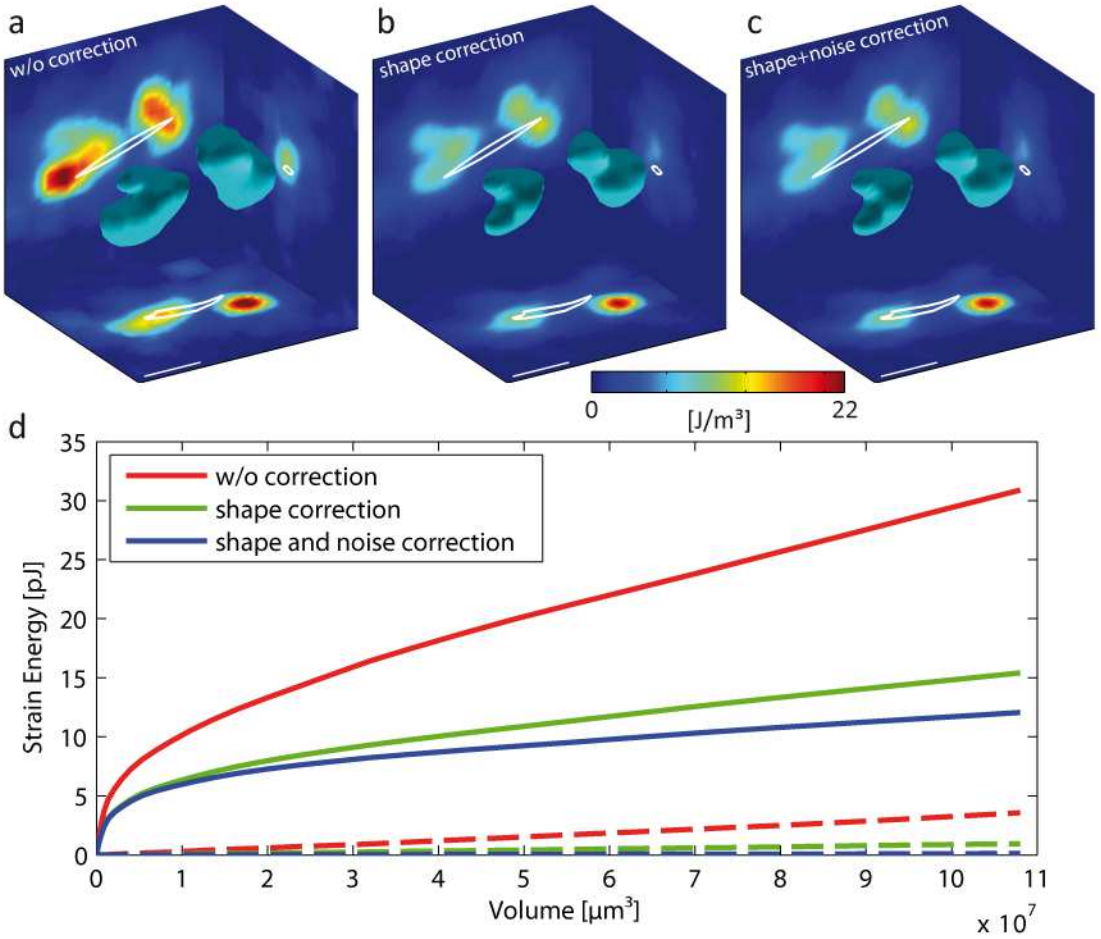

Figure S6: Noise correction of strain energy density. a: strain energy density distribution around an A-125 lung carcinoma cell without any correction, b: after eliminating tetrahedral elements with shape quality factor q<0.5, c: after additional noise subtraction according to Eq. (15). d: Integration of strain energy density over increasingly larger volumes shows the importance of shape and noise correction for recovering the strain energy. Here, the solid lines correspond to strain energy measurements of the cell shown in a-c; the dashed lines to the strain energy measured in a gel without cells.

Strain energy convergence

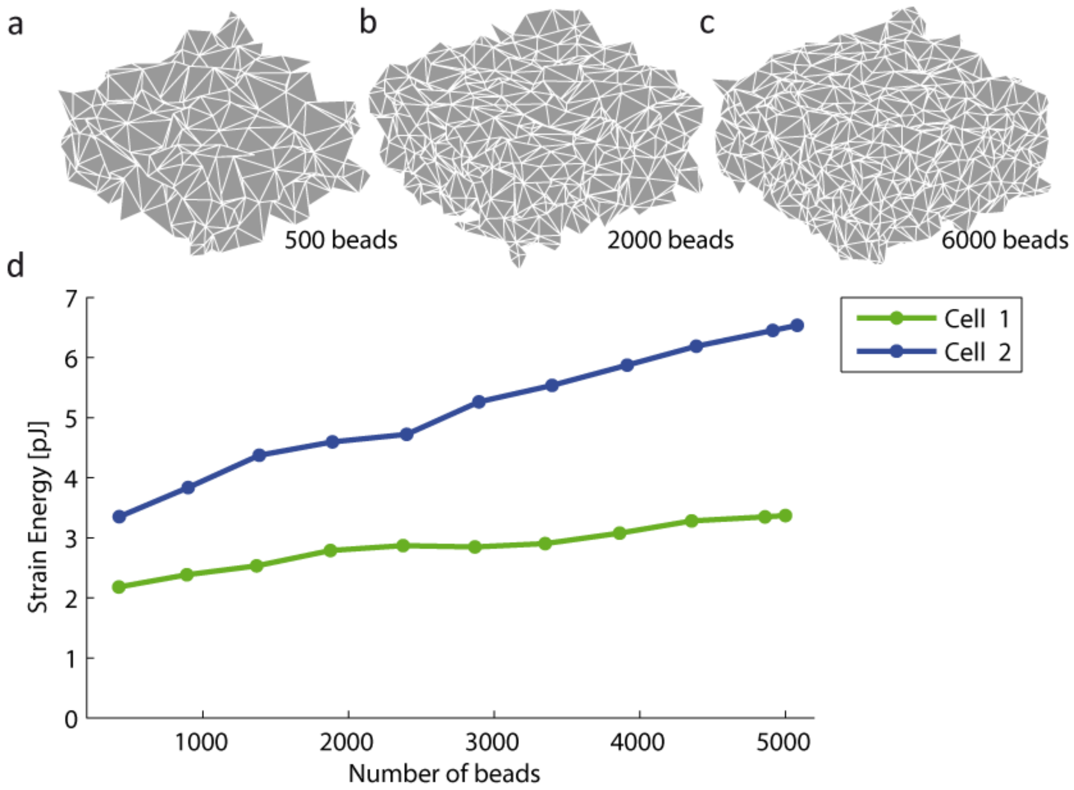

Figure S7: Strain energy convergence. Strain energy in a fixed volume around two representative cells evaluated on a subset of beads, i.e. the number of beads in an experimental data set was systematically reduced (a, b, and c show resulting finite element meshes) to obtain the curves in d.

In a linear continuum material and under noise-free conditions, the accuracy for estimating the strain energy is limited only by the interpolation accuracy within the tetrahedral elements. Using an increasing number of beads would enable mapping out increasingly finer details of the strain energy distribution until eventually convergent results are obtained. In practice, there exists an upper bound for the number of beads that can be dispersed in the gel, because of the fibrous nature of the material and due to optical effects, e.g. blur from out-of-focus beads in a conventional epifluorescence microscope.

To estimate the sensitivity of the strain energy to the number of beads used, we performed our analysis on a subset of beads with varying densities as illustrated in Fig. S7. A number of beads were randomly drawn from the measured data set, and the strain energy was computed on this subset. As expected, the strain energy around a cell increases with the number of beads (solid curves). With a 10-fold higher bead density, the total strain energy approximately doubled. The strain energy did not saturate even at the highest bead density, however, suggesting that the method underestimates the true strain energy, although part of the raise in strain energy with increasing bead density can be explained by an incomplete noise correction.

Experimental verification of a continuum mechanical description of collagen gels

To test whether the continuum assumption of the collagen gel holds at the length scale of typical bead-to-bead distances (24 µm), we deformed the gel with a superparamagnetic bead that was embedded in the collagen gel and laterally forced with magnetic tweezers. The collagen gel was prepared according to our protocol but with the addition of superparamagnetic beads of 4.5 µm

diameter. A 20 µl droplet of the mixture was placed on a cell culture dish and allowed to polymerize upside down so that the magnetic beads drifted to the surface of the droplet. After polymerization, we recorded z-stacks through the gel before and after the application of a 58 nN force acting on an isolated magnetic bead. Force calibration of the magnetic tweezer setup was performed as explained previously[5]. The displacement vectors of the fluorescent beads surrounding the magnetic bead between the force-on and force-off states are shown in Fig. S8a-b. The displacements in a continuum linear elastic half-space due to a point force acting on the surface are given by the Boussinesq solution[6] and were fitted to the measured displacements of the fluorescent beads. The measured bead displacements closely follow the Boussinesq displacement field (Fig. S8a), confirming that the deformation field of the collagen gel can be described by a continuum approach. The Poisson's ratio enters the fit as an independent parameter and was found to be 0.35; this value was used for computing the strain energy in all cell experiments.

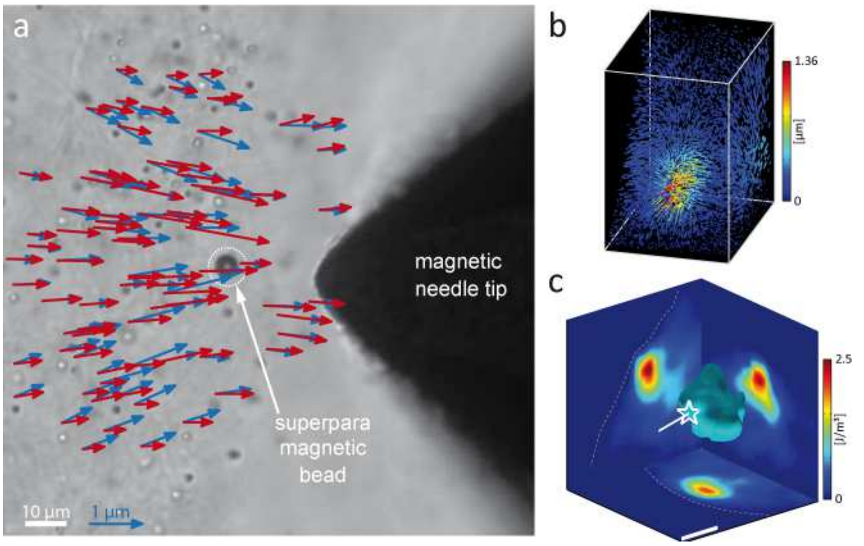

Figure S8: Displacements and strain energy density around a deflected superparamagnetic bead. a: Measured (blue) and fitted (red) projected displacements of fluorescent beads embedded in a collagen gel in the vicinity of a superparamagnetic bead. The gel was deformed with a force of 58 nN acting on the magnetic bead. b: Measured displacements of the fluorescent beads in the collagen drop between the force-on and force-off states. c: Corresponding strain energy density distribution around the magnetic bead indicated by the white star. The direction of the applied force is shown by the white line.

Experimental verification of strain energy computation

To quantitatively assess the accuracy of the measured strain energy, we computed the strain energy in the collagen droplet surrounding the magnetic bead between the force-on and force-off states. The strain energy density around the magnetic bead is shown in Fig. S8c and falls off rapidly with increasing distance from the magnetic bead, as expected. The integrated total strain energy stored in the gel was measured to be 0.08 pJ. This value agrees well with the expected strain energy of 0.1 pJ, calculated from the work done on the magnetic bead as it moved towards the magnetic needle (force acting on the bead times bead displacement times 0.5 according to Clapeyron's theorem[7]).

Furthermore, we carried out a simple indentation experiment. A spherical steel ball of 100 µm diameter was placed on the surface of a collagen gel. The ball indented the gel by 36.1 µm due to a gravitational force of 35.4 nN. The displacements of the measured bead positions between the undeformed and deformed positions are plotted in Fig. S9b. Bead displacements are projected on a cylindrical coordinate system with the axis going through the center of the ball. Because bead

displacements larger than 10 µm could not be reliably tracked, we determined the strain energy in a donut shaped subvolume of the gel as indicated by the red outline in Fig. S9b and Fig. S9c. We have furthermore calculated the expected strain energy of the same volume from a finite element simulation of the experiment as illustrated in Fig. S9a, with the measured indentation depth as a prescribed condition for the displacement of the ball. Due to symmetry, the finite element model geometry was constructed with a planar section through the center of the ball together with cylindrically symmetric boundary conditions and fixed walls. Contact between the ball and the gel was modeled with a no-slip condition. The simulation was carried out with Abaqus (SIMULIA, Providence, RI, USA). The displacement results of the simulation were then transferred to the 3D geometry of the experiment and the strain energy was calculated with our algorithm. The result of the simulation was 1.5 pJ and agrees well with that of the experimentally obtained strain energy of 1.6 pJ.

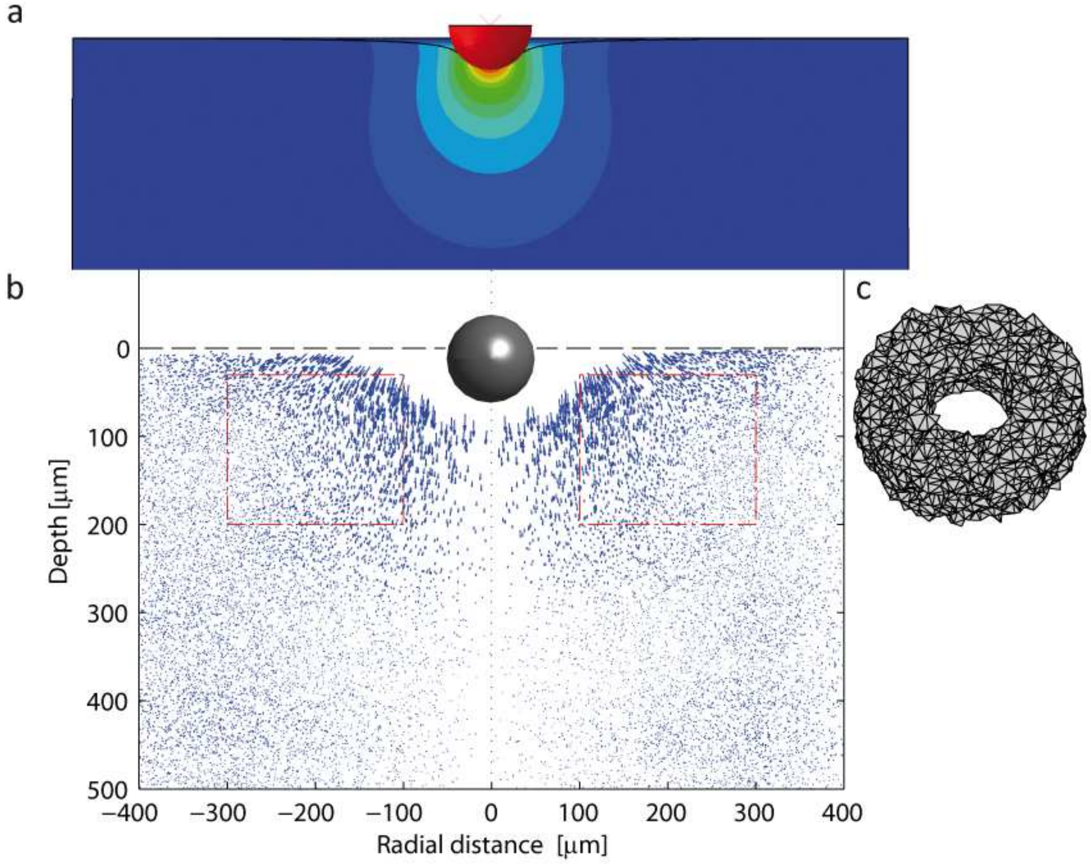

Figure S9: Experimental verification of the strain energy measurement by an indentation experiment with a steel ball. a: Finite Element simulation of the experiment. b: Measured displacements projected on a cylindrical coordinate system with the z axis going through the center of the steel ball. c: The strain energy was evaluated in a donut shaped subvolume as indicated by the red lines in b)

Total strain energy, cell shape anisotropy and strain energy anisotropy follow a log-normal distribution

Cumulative probabilities of all measured cell data were evaluated and are shown in Fig. S10 for strain energy values, in Fig. S11 for cell shape, and in Fig. S12 for strain energy anisotropy indices. The latter two measures are bounded by 1 and this bias was taken into account before computation. The cumulative probability assuming an underlying log-normal distribution is plotted for comparison showing that the distribution of total strain energy among individual cells closely follows a log-normal distribution.

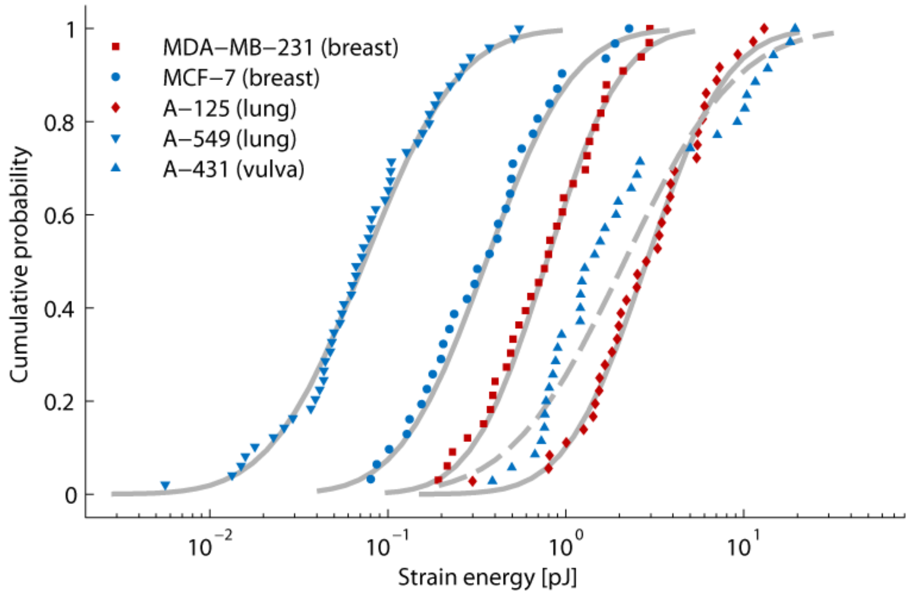

Figure S10: Cumulative probabilities of total strain energy for various carcinoma cell lines. Each data point corresponds to a measurement from a different cell. The lines are cumulative probabilities from a best-fit log-normal distribution. With the exception of A-431 cells, cellular strain energy data follow a log-normal distribution.

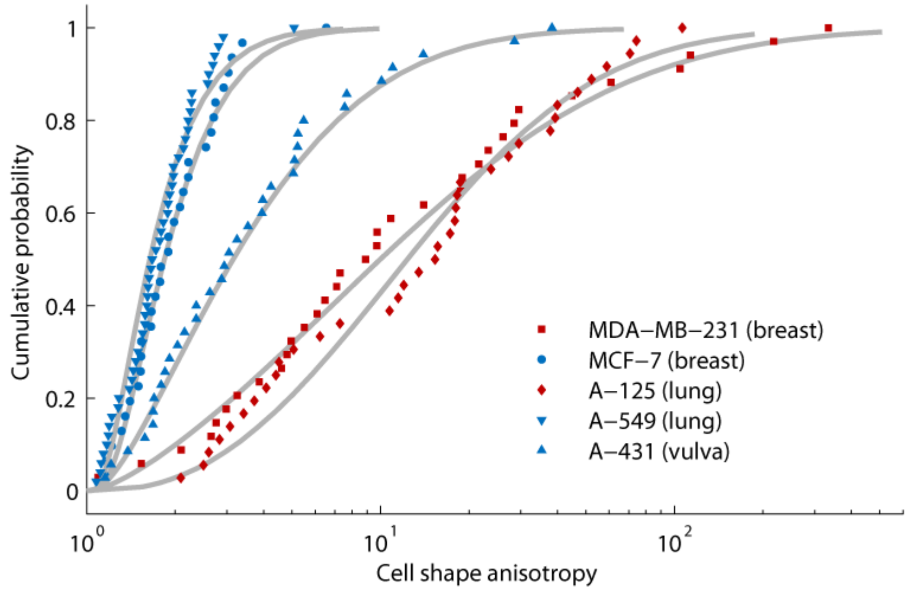

Figure S11: Cumulative probabilities of cell shape anisotropy for various carcinoma cell lines. Each data point corresponds to a measurement from a different cell. The lines are cumulative probabilities from a best-fit log-normal distribution.

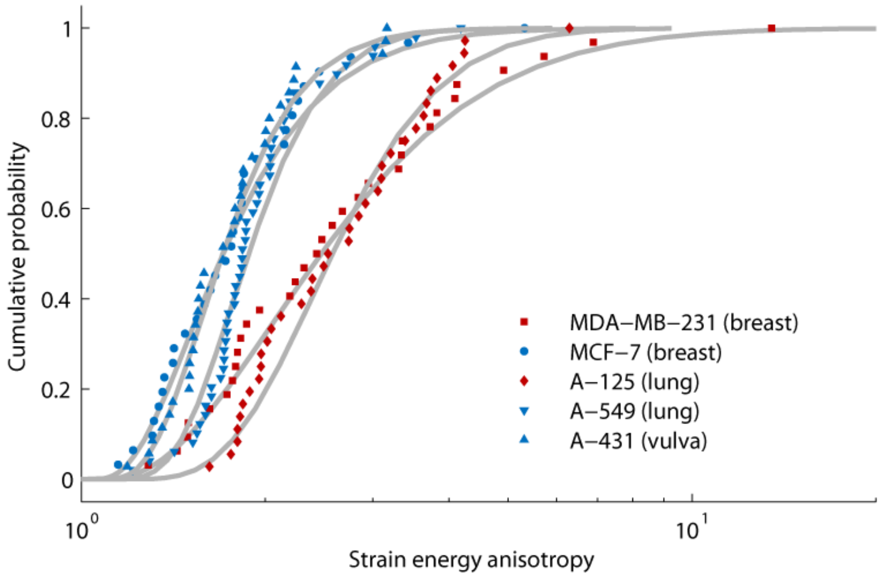

Figure S12: Cumulative probabilities of strain energy density anisotropy for various carcinoma cell lines. Each data point corresponds to a measurement from a different cell. The lines are cumulative probabilities from a best-fit log-normal distribution.

Time-lapse measurements of strain energy in 3D collagen gels

The technique for measuring the 3-D strain energy can be readily combined with time-lapse recordings by repeatedly taking image stacks around cells embedded in 3D collagen gels. A last stack imaged after the release of cell tension (e.g. by treatment with cytochalasin D) serves as the reference stack for obtaining the bead positions at the force-free state. With this approach, further insight can be gained into the dynamics of cellular traction generation during cell migration.

In the following we present two examples, one of a highly contractile yet non-invasive vulva carcinoma cell (A-431), and one of an invading breast carcinoma cell (MDA-MB-231). In both cases, cells have been mixed to the collagen solution prior to polymerization and allowed to spread for 24h as described previously. Image stacks were then taken at time intervals of 15 min and 20 min, respectively. To reduce phototoxicity, light intensities were minimized as far as possible, and the exposure shutter was closed between imaging.

During the measurement period of 165 min before force release, the vulva carcinoma cell kept the surrounding collagen matrix under high tension at all times but the total strain energy fluctuated by up to 25% around the time average (Fig. 4a).

The fluctuations of total strain energy are caused by fluctuations of strain energy density that varied both temporally and spatially around the cell. An example of the temporal and spatial distribution of strain energy fluctuations is shown in Fig. S13-S14 and Movie S3. The cell moved during the measurement period in a random manner but remained within a few µm of its starting position. In concert with this random cell movement, the strain energy density distribution around the cell shifted as well. This is illustrated by contour lines of the local change in strain energy density between two time steps t_1 and t_2, computed as

$$\Delta\tilde{U} = \frac{\overline{U}(t_2) - \overline{U}(t_1)}{\overline{U}(t_2)}\ .$$

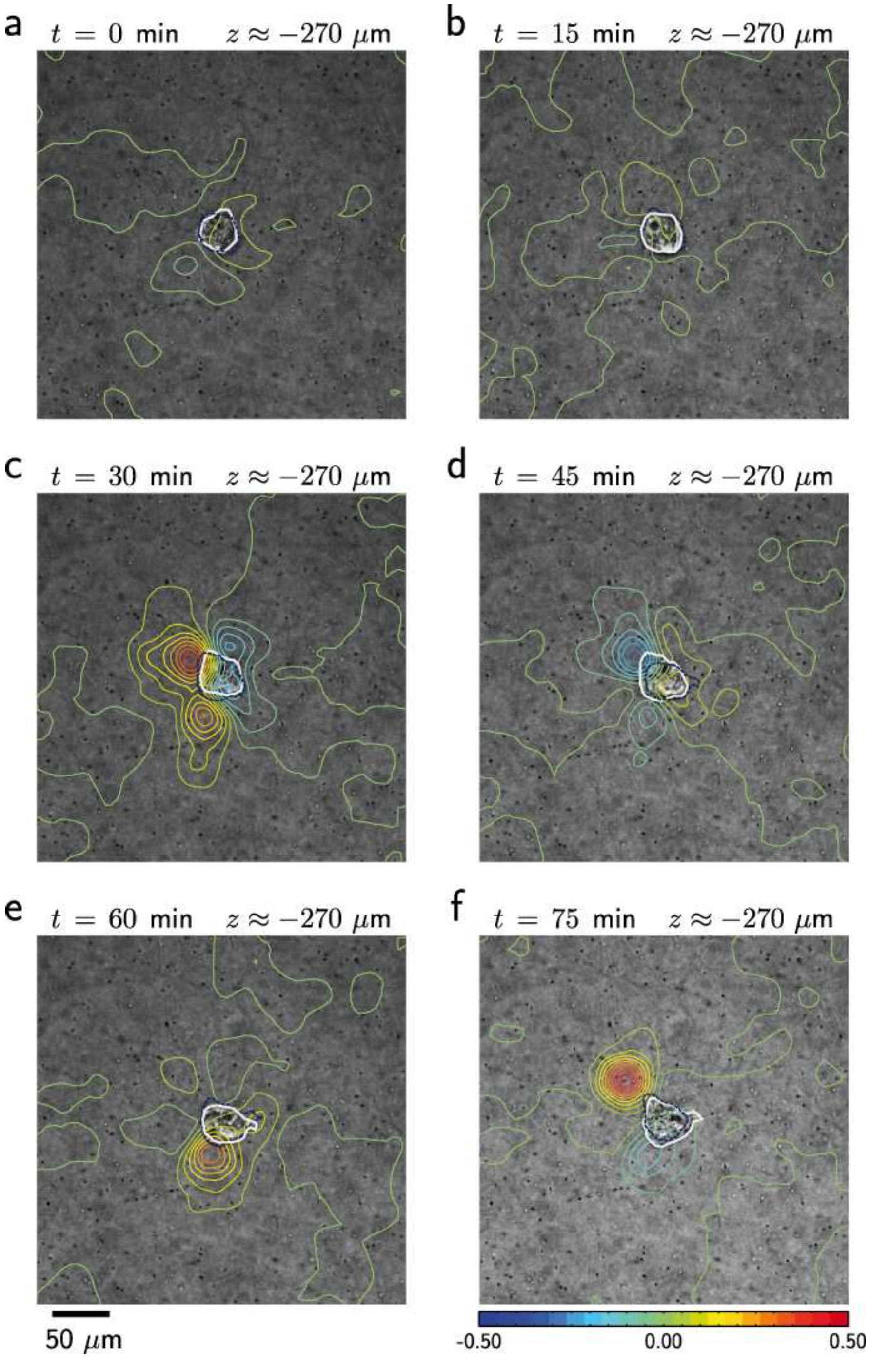

Figure S13: Time-lapse measurement of a non-invasive vulva carcinoma cell. Time series of brightfield images at the location of a non-invasive vulva carcinoma cell (A-431) embedded in a 3D collagen gel. Superimposed are contour lines encoding relative changes in strain energy density between two subsequent time steps, indicating how the distribution of strain energy density shifts around the proximity of the cell during the measurement. The cell shapes at the first and second time step are outlined in white and dark blue, respectively, to indicate the changes in cell position.

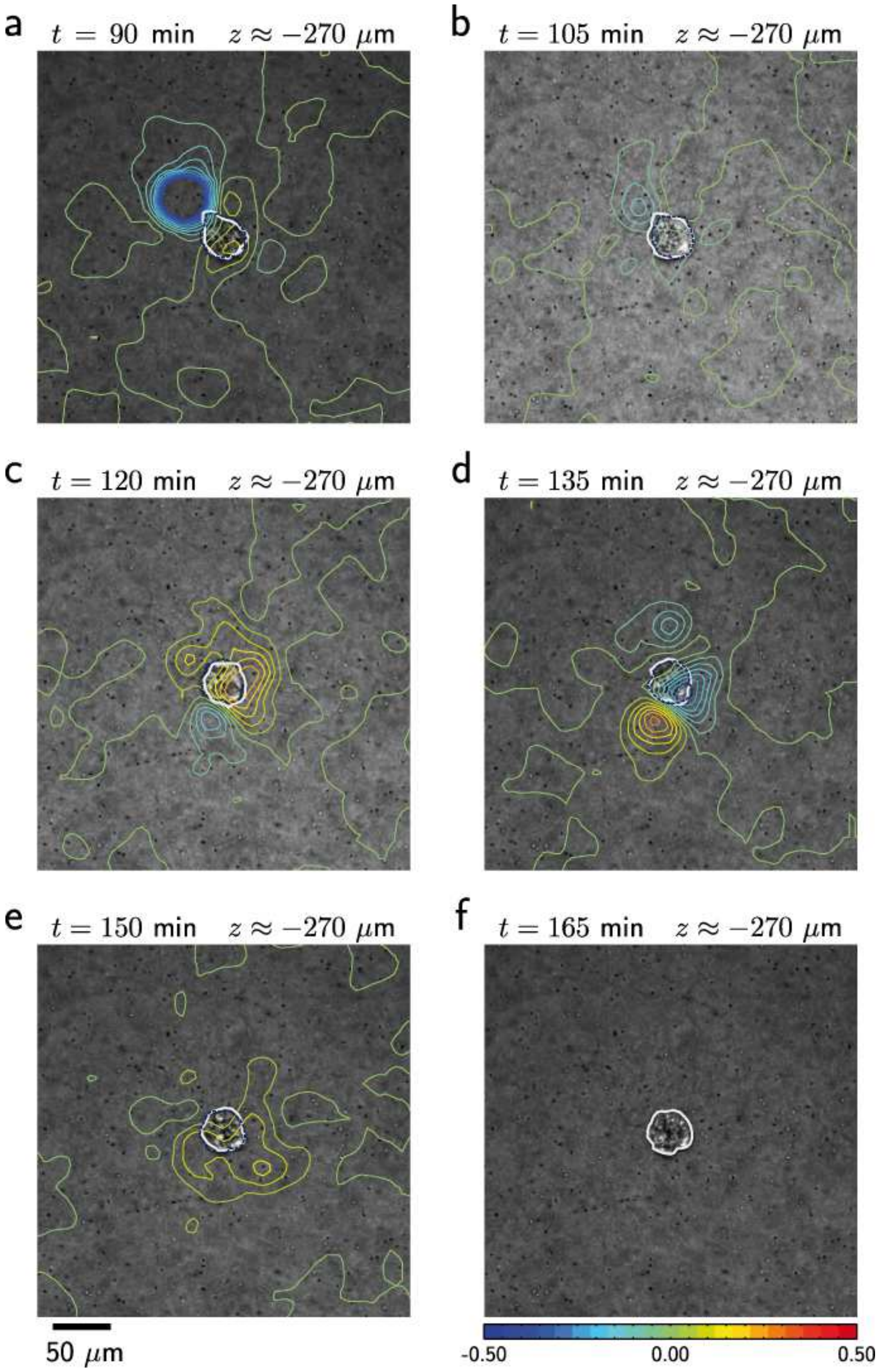

Figure S14: Continuation of Fig. S13

Negative values with corresponding contour lines towards the blue end of the spectrum point to regions where tension is relaxed between successive time steps. Contour lines towards the red end of the spectrum point to regions where tension is increased between successive time steps.

In the case of the non-invasive vulva carcinoma cell, we observe periods of comparatively small changes (Fig.S13a,b and Fig.S14b,e) and periods of comparatively large changes in the strain energy density distribution (Fig.S13c-f and Fig.S14a,c,d). It is interesting to note that areas of increasing tension at one time step generally turn into areas of decreasing tension in the following time step. In summary, this non-invasive carcinoma cell keeps the collagen gel under high tension all time steps,

with temporally fluctuating regions of high and low strain energy density that shift the cell around in a random, non-persistent fashion during seemingly uncoordinated cycles of tension generation and release.

In the second example, an invasive breast carcinoma cell also keeps the collagen gel under high tension during the measurement period of 360 min (Fig.4b), but with larger fluctuations (more than 50% around the time average) compared to the non-invasive cell. Moreover, the cell migrated through the collagen in all three spatial directions by a distance spanning more than 100 µm (Fig. S15-S17 and Movie S4).

Compared to the non-invasive cell, the temporal and spatial pattern of strain energy density fluctuations shows two striking differences. First, the changes in strain energy density are considerably more persistent in time, meaning that a local increase during one time step is usually not followed by a corresponding decrease over the following time step. Second, local changes in strain energy density often preceded shape changes of the cell and are followed by a cell movement in the same direction for positive strain energy changes, or in the opposite direction for negative changes.

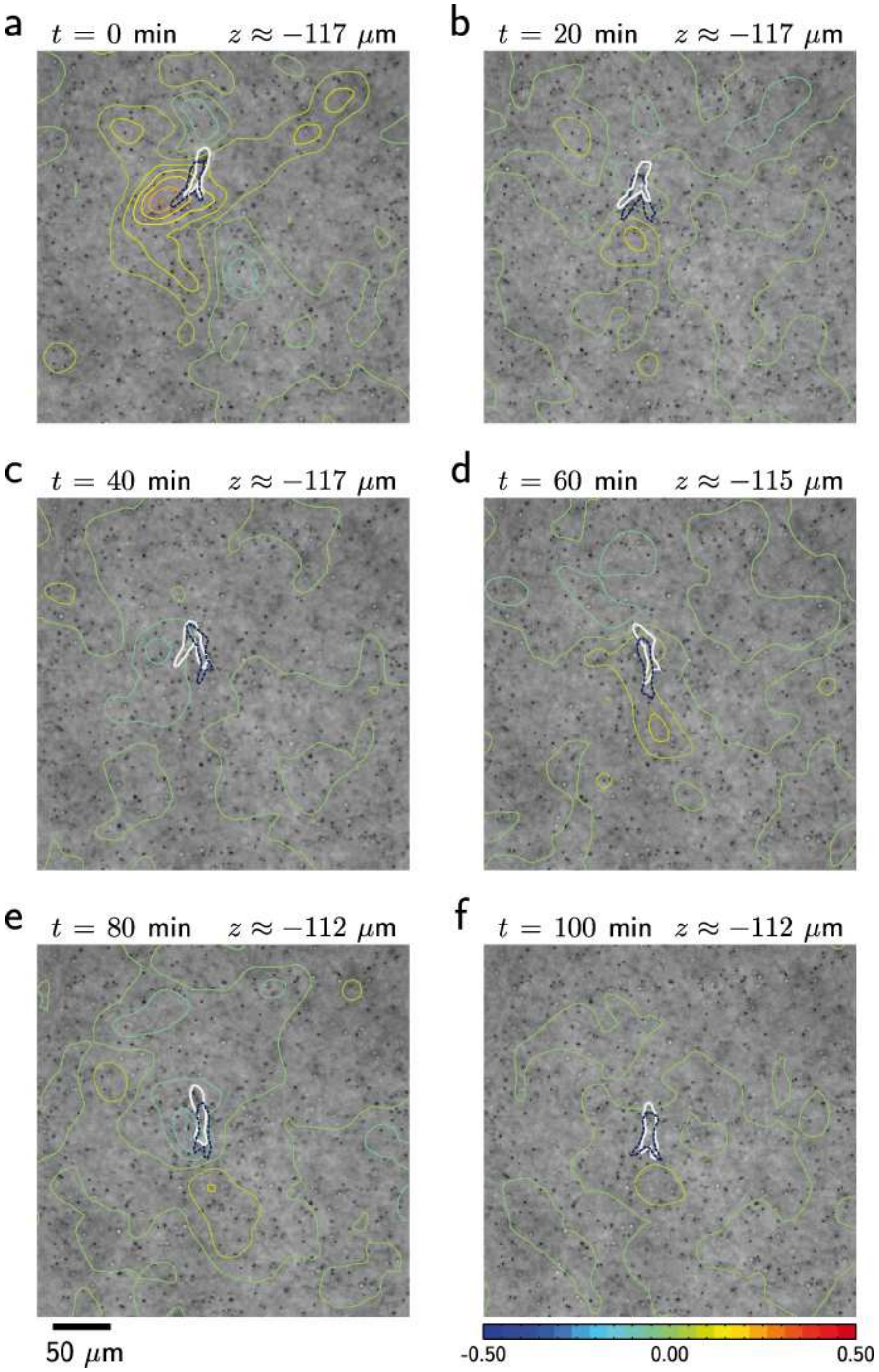

Figure S15: Time-lapse measurement of an invasive breast carcinoma cell. Time series of brightfield images at the location of an invasive breast carcinoma cell (MDA-MD-231) embedded in a 3D collagen gel. Superimposed are contour lines encoding relative changes in strain energy density between two subsequent time steps, indicating how the distribution of strain energy density shifts around the proximity of the cell during invasion. The cell shapes at the first and second time step are outlined in white and dark blue, respectively, to indicate the changes in cell position.

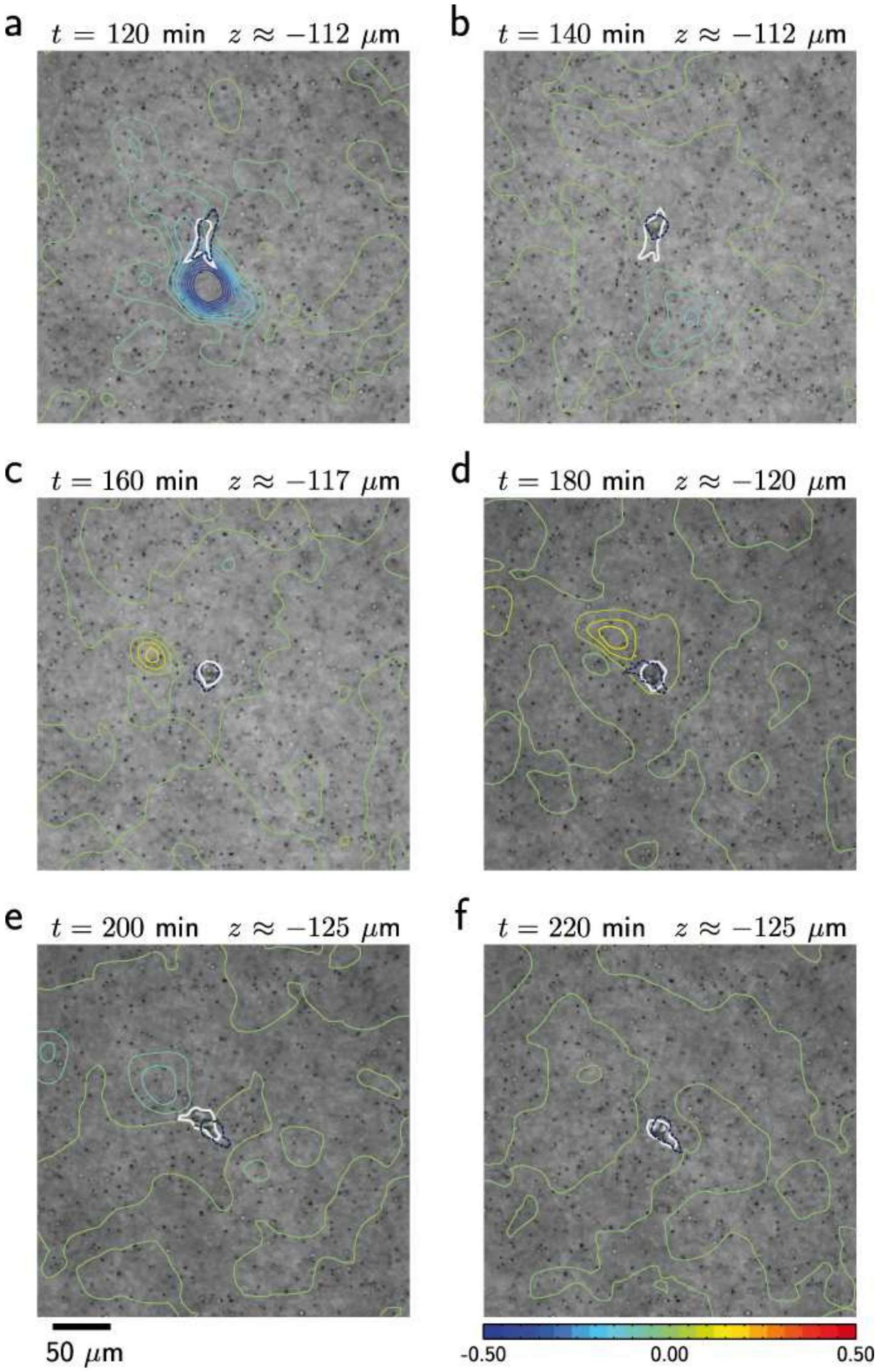

Figure S16: Continuation of Fig.S15

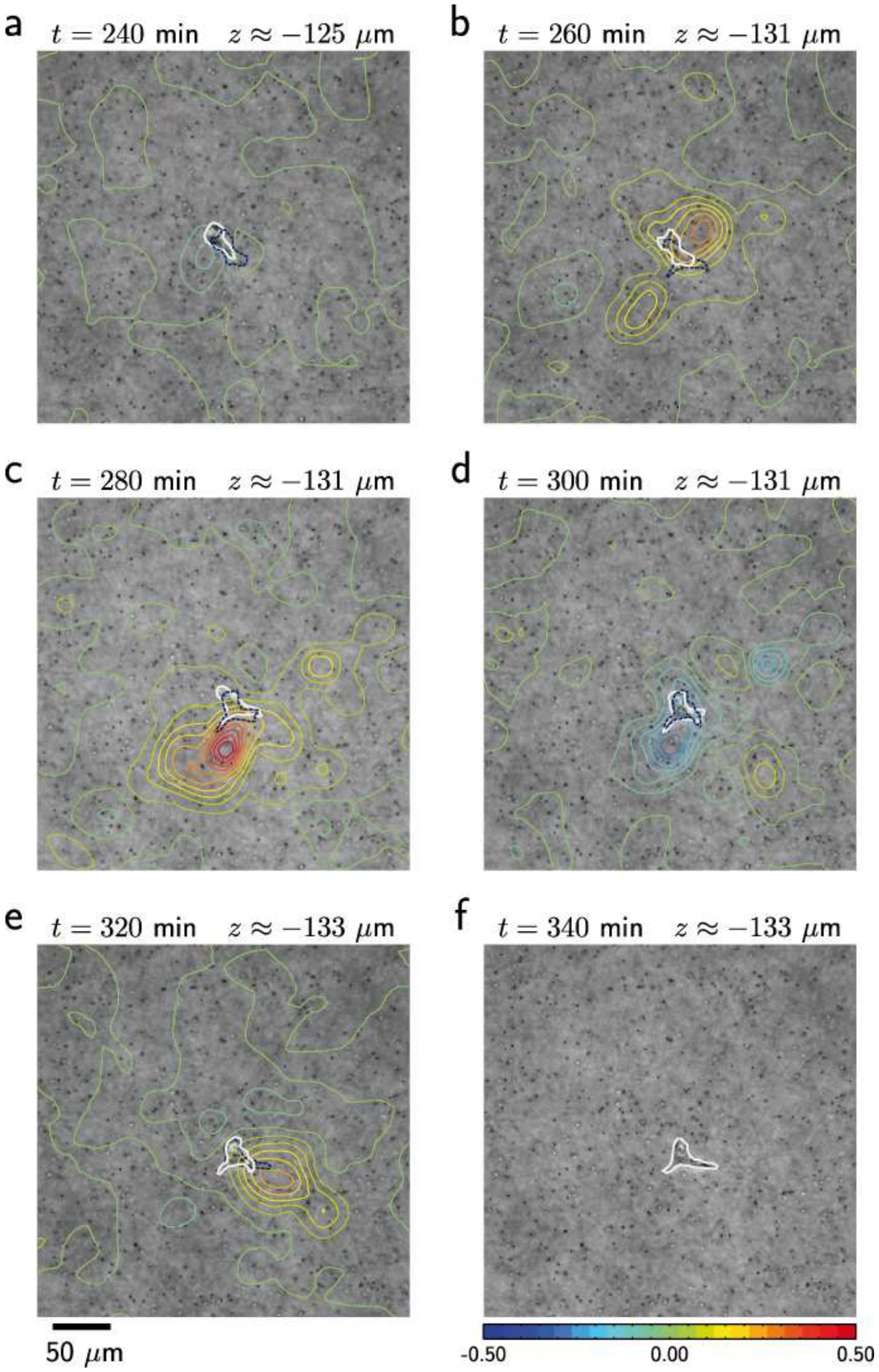

Figure S17: Continuation of Fig.S16

Software

The algorithms presented in this paper implemented in MATLAB (MathWorks, Natick, MA, USA) are available as Supporting Information File S1.

References

1. Mickel W, Muenster S, Jawerth LM, Vader DA, Weitz DA, et al. (2008) Robust Pore Size Analysis of Filamentous Networks From 3D Confocal Microscopy. Biophys J 95: 6072-6080.

2. Zienkiewicz OC, Taylor RL, Zhu JZ (2005) The Finite Element Method: Its Basis and Fundamentals: Butterworth Heinemann.

3. Barber CB (1996) The Quickhull Algorithm for Convex Hulls. ACM Trans Math Software 22: 469-483.

4. Alberty J, Carstensen C, Funken SA, Klose R (2002) Matlab Implementation of the Finite Element Method in Elasticity. Computing 69: 239-263.

5. Kollmannsberger P, Fabry B (2007) High-Force Magnetic Tweezers with Force Feedback for Biological Applications. Rev Sci Instrum 78: 114301-114301-114306.

6. Landau LD, Pitaevskii LP, Lifshitz EM, Kosevich AM (1986) Theory of Elasticity, Course of Theoretical Physics, Volume 7: Butterworth-Heinemann.

7. Sadd MH (2009) Elasticity: Theory, Applications, and Numerics: Academic Press.
