# Supplementary material for: 3D Traction Forces in Cancer Cell Invasion
Source: PLoS One. 2012 Mar 30;7(3):e33476. doi: 10.1371/journal.pone.0033476 (PMC3316584; doi:10.1371/journal.pone.0033476)
Supplement: File S1 — The MATLAB (MathWorks, Natick, MA, USA) source code of all necessary programs to carry out measurements of strain energy in 3D collagen gels. (ZIP) [file pone.0033476.s023.zip › SoftwareDescription.pdf]

Software for 3D Strain Energy Measurements

Included with this paper are MATLAB implementations of the algorithms presented together with an example data set. In this section, we give a brief description of the MATLAB programs and a short tutorial on their usage.

Description of the programs

Prerequisites

The algorithms work on 3-D image stacks stored as individual 2-D images. Two fluorescence image stacks are required for the measurement of strain energy, one representing the deformed, the other representing the undeformed gel configuration. Corresponding bright field image stacks are useful to locate the cell and to observe cellular deformation. The following naming convention is used by default but can be adjusted as described below. Fluorescent file names start with 'FLframe_', bright field files with 'BFframe_'. This is followed by the z location within the stack, counted from 0 at the top towards the bottom of the stack, and a stack count starting from 1. For instance, 'FLframe_z042_000002.jpg' refers to the 43rd z slice at the second image stack (normally this is the image stack after relaxation) recorded in a fluorescent mode and stored in lossless JPEG format.

Units

The algorithms use SI units, except for the unit measuring lengths, where numbers are given in µm. Derived units change accordingly, e.g. the strain energy results are in units of pJ:

$$1\,\mathrm{pJ} = 1\,\mathrm{pNm} = 1\mathrm{p}\frac{\mathrm{kg\,m}}{\mathrm{s}^2}\mathrm{m} = 1\frac{\mathrm{kg}\,(\mu\mathrm{m})^2}{s^2}$$

`getStainEnergyAssayConfig`

This is the main configuration file for all algorithms and is the place to make adjustments such as names of output files etc.

`getFLFileName`

This function returns the file name of an optical fluorescence slice and can be modified according to the file naming convention used. The function takes 2 arguments: The first is the z location of the image measured from the top (0), with increasing positive integers for increasing depths. The second argument is the stack count.

`getBFFileName`

This function is similar to the above, except it returns file names for bright field slices.

`startTracking`

starts the bead tracking algorithm.

`startStrainEnergyComputation`

starts the computation of strain energy.

`trackBeads3D`
the 3D bead tracking algorithm.

`strainEnergyComputation`
computes the strain energy from the tracked bead positions.

`browseStack`
Call this function to navigate through the images in the z- and time dimensions. The function can also be used to load tracked bead data to allow visual inspection of the tracking results. It furthermore can be used for manually outlining the cell contour. For a list of options enter the command '`help browseStack`'.

`sedensIsoProjectionFigure`
This function creates plots of strain energy density.

`strainEnergyResults`
Use this script for post-processing of strain energy results. It creates plots of displacements and strain energy density around a cell and computes the integrated strain energy.

Tutorial

Step 0: Matlab and software configuration
Start Matlab and add the path containing the strain energy software to the Matlab search path, e.g. by entering the command '`addpath('C:\your_path_here')`'.

Open the file '`getStrainEnergyAssayConfig`' in the editor and adjust all values of the configuration variables (documented in the file) to accommodate your setup.

Step 1: Locate the cell
In Matlab, set the current directory to the directory containing the images of a measurement. Call '`browseStack`' and navigate through the images until you find the cell. Make sure that the stack you currently view corresponds to the undeformed configuration. Press 'o' to enter the manual outlining mode and create a planar outline of the cell. Double click when you are done. The program then calculates the cell center, direction and area and saves it in the file 'cell_shape_XXX.mat' where 'XXX' corresponds to the current stack count. This file is later used to re-center the coordinate system, such that the cell is located at the origin with the cell direction defining the x-axis.

Step 2: Bead tracking
Call the '`startTracking`' function to initiate the bead tracking algorithm. The user will be prompted to enter a directory containing the images. After that, the bead tracking will start.

Step 3: Computation of strain energy
Call the '`startStrainEnergyComputation`' function to calculate the strain energy.

Step 4: Post-process strain energy results
Edit the '`strainEnergyResults`' script to adjust post-processing parameter such as output filenames and plot dimension etc. (The parameters are documented within the script.)

Run the script. It generates plots of strain energy density and displacements around the cell and computes the integrated strain energy.
